# Validation of a Custom Next-Generation Sequencing Assay for Cystic Fibrosis Newborn Screening

**DOI:** 10.3390/ijns7040073

**Published:** 2021-11-02

**Authors:** Robert J. Sicko, Colleen F. Stevens, Erin E. Hughes, Melissa Leisner, Helen Ling, Carlos A. Saavedra-Matiz, Michele Caggana, Denise M. Kay

**Affiliations:** 1 New York State Newborn Screening Program, Division of Genetics, Wadsworth Center, New York State Department of Health, Albany, NY 12208, USA; robert.sicko@health.ny.gov (R.J.S.); colleen.stevens@health.ny.gov (C.F.S.); erin.hughes@health.ny.gov (E.E.H.); carlos.saavedra@health.ny.gov (C.A.S.-M.); michele.caggana@health.ny.gov (M.C.); 2 Applied Genomics Technologies Cluster, Wadsworth Center, New York State Department of Health, Albany, NY 12208, USA; melissa.leisner@health.ny.gov (M.L.); helen.ling@health.ny.gov (H.L.)

**Keywords:** cystic fibrosis (CF), next-generation sequencing (NGS), newborn screening (NBS), IRT-DNA-SEQ algorithm

## Abstract

Newborn screening (NBS) for Cystic Fibrosis (CF) is associated with improved outcomes. All US states screen for CF; however, CF NBS algorithms have high false positive (FP) rates. In New York State (NYS), the positive predictive value of CF NBS improved from 3.7% to 25.2% following the implementation of a three-tier IRT-DNA-SEQ approach using commercially available tests. Here we describe a modification of the NYS CF NBS algorithm via transition to a new custom next-generation sequencing (NGS) platform for more comprehensive cystic fibrosis transmembrane conductance regulator (*CFTR*) gene analysis. After full gene sequencing, a tiered strategy is used to first analyze only a specific panel of 338 clinically relevant *CFTR* variants (second-tier), followed by unblinding of all sequence variants and bioinformatic assessment of deletions/duplications in a subset of samples requiring third-tier analysis. We demonstrate the analytical and clinical validity of the assay and the feasibility of use in the NBS setting. The custom assay has streamlined our molecular workflow, increased throughput, and allows for bioinformatic customization of second-tier variant panel content. NBS aims to identify those infants with the highest disease risk. Technological molecular improvements can be applied to NBS algorithms to reduce the burden of FP referrals without loss of sensitivity.

## 1. Introduction

Cystic Fibrosis (CF; OMIM # 219700) is an autosomal recessive condition characterized by abnormal secretion of electrolytes and fluid across epithelial membranes of most exocrine organs. Clinical symptoms of CF include persistent cough; frequent lung infections including pneumonia or bronchitis; wheezing or shortness of breath; poor growth or weight gain; frequent greasy, bulky stools or difficulty with bowel movements; salty-tasting skin; male infertility. Identification of infants with CF via newborn screening (NBS) is cost-effective and associated with improved outcomes [1].

CF is one of the more common conditions screened at birth. While pan-ethnic, the incidence varies by race/ethnicity and by geographic region. The overall incidence of CF in New York State (NYS) was 1 in 5700 between 2007 and 2012 [2]. Homozygous or compound heterozygous variants in the cystic fibrosis transmembrane conductance regulator (*CFTR*) gene cause CF. NYS started screening for CF in October 2002 using a two-tier IRT-DNA algorithm consisting of first-tier immunoreactive trypsinogen (IRT) screening, followed by targeted screening using a *CFTR* variant panel for infants with IRT values in the daily top 5%. Infants in the top 5% daily IRT with one or more panel variants and infants with no variants but ultra-high/very high IRT (VHIRT) were referred for sweat testing [2,3]. Targeted variant panels improve the specificity of CF NBS algorithms. However, the CF false positive rate was still high in NYS because carriers and infants with VHIRT were referred for diagnostic evaluation since only a limited genotyping panel was utilized and rare CF-causing variants could not be tested [3].

The California NBS program was the first to introduce an IRT-DNA-SEQ CF algorithm including third-tier *CFTR* gene sequencing, in 2007 [4,5]. Sequencing was performed in specimens with one variant identified via a targeted panel, by an outside clinical laboratory. The algorithm reduced the false positive rate and increased NBS sensitivity, especially in non-White populations, yielding a 34% positive predictive value (PPV) overall [5,6]. Studies have since demonstrated the feasibility of panel-based or comprehensive *CFTR* gene analysis in dried blood spots (DBS) using NGS [7,8]. The Wisconsin NBS program also conducts post-referral *CFTR* gene sequencing via NGS in a subset of infants with one panel variant after a positive sweat chloride test (>30 mmol/L) [9]. In this context, the targeted panel is used to identify rare *CFTR* variants in infants with intermediate or elevated sweat chloride but is not used for NBS decision-making and therefore does not directly influence the false-positive screen rate.

In 2017, a three-tier IRT-DNA-SEQ algorithm was implemented by the NYS NBS program after validation of an FDA-cleared CF NGS kit for use with DBS. Inclusion of complete *CFTR* sequencing permitted referral for only the infants at highest risk for CF (i.e., those with two clinically relevant variants). Elimination of referral of CF carriers and infants with VHIRT resulted in an 83.1% reduction in referrals and a nearly seven-fold increase in positive predictive value (3.7% to 25.2%) during the first year of IRT-DNA-SEQ in NYS. Elimination of referral for infants without two variants was only possible via comprehensive genetic analysis, which is necessary to maintain high NBS sensitivity in the diverse NYS population. Prior to the advent of NGS technology, comprehensive *CFTR* gene analysis would have required Sanger sequencing, which was too expensive, labor-intensive, and time-consuming for routine NBS.

Here, we demonstrate a further improvement of the NYS CF NBS algorithm, with the implementation of a new *CFTR* molecular assay utilizing NGS technology that includes a customizable second-tier panel and combines second- and third-tier testing onto a single platform, streamlining workflows.

## 2. Materials and Methods

### 2.1. Initial NYS CF IRT-DNA-SEQ Algorithm (1 December 2017–30 June 2019)

**Figure 1:**
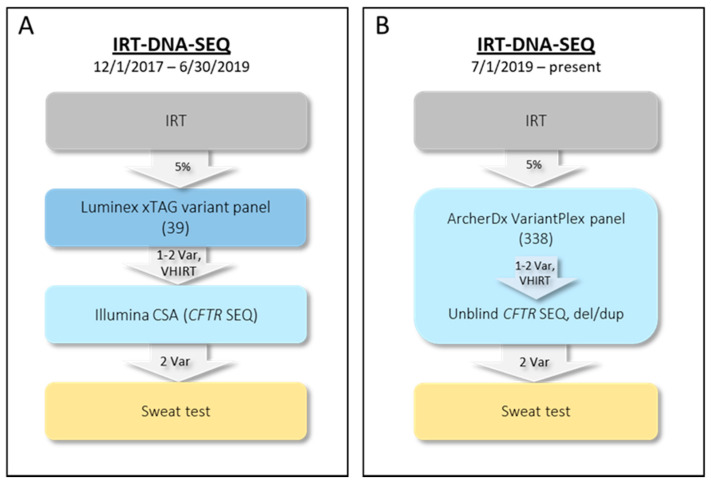
NYS IRT-DNA-SEQ CF algorithm. (**Panel A**) shows the initial three-tier NYS CF algorithm using commercially available tests, and (**Panel B**) shows the current algorithm using the custom assay. *IRT—*immunoreactive trypsinogen; *VHIRT*—ultra-high/very high IRT, 0 variants; *Var*—clinically relevant variant; *CSA*—Illumina MiSeqDx Cystic Fibrosis Clinical Sequencing Assay; *SEQ—*full *CFTR* sequencing; *del/dup*—deletion/duplication analysis.

The two-tier NYS IRT-DNA algorithm utilized between 2002 and 2017 has been described [2,3]. A three-tier IRT-DNA-SEQ algorithm was implemented in 2017. From 1 December 2017 through 30 June 2019, second-tier molecular screening (DNA) for 39 *CFTR* variants was performed on specimens in the IRT daily top 5% using the Luminex xTAG CF39v2 kit (Figure 1A). Infants with at least one panel variant or no panel variants but VHIRT (top 0.1% calculated over a 10-day interval) were reflexed to third-tier testing (SEQ). Since September 2019, IRT levels have been tested using the PerkinElmer GSP Neonatal IRT kit. The median daily 5% cutoff value is 53 ng/mL (range 44–65 ng/mL), and the median 10-day 0.1% cutoff value is 117 (range 90–166 ng/mL). The Illumina MiSeqDx Cystic Fibrosis Clinical Sequencing Assay (CSA) with supplemental deletion analysis of exons 2, 14 (13), and 20 (17b; legacy *CFTR* nomenclature listed in parentheses throughout, if different from HGVS nomenclature) was used for third-tier molecular analysis. Infants with two *CFTR* variants of potential clinical significance identified via the DNA panel or SEQ were referred to a NYS CF Specialty Care Center for diagnostic sweat chloride testing and evaluation. Rather than referral, infants with only one *CFTR* variant were issued a report with a recommendation for genetic counseling, and those with no variants were released as screen negative.

### 2.2. Current NYS CF IRT-DNA-SEQ Algorithm (1 July 2019–Present)

To streamline the molecular workflow and to expand the targeted panel to include more CF-causing variants, we sought to develop and validate a new molecular CF assay. The new algorithm still follows the IRT-DNA-SEQ approach, but rather than using separate second- and third-tier assays, a single assay with a tiered analysis strategy is utilized (Figure 1B). The custom VariantPlex *CFTR* NGS assay (henceforth, Archer CF assay) was designed as a collaboration between the NYS NBS program and ArcherDx. ArcherDx VariantPlex assays use anchored multiplex PCR (AMP) to amplify regions of interest using unidirectional gene-specific primers (GSPs). Adapters that contain both molecular barcodes (MBCs) and sample indices permit quantitative multiplex data analysis, read de-duplication, and accurate variant calling. MBCs also permit screening for exon deletions and duplications (del/dup) using molecular counting, increasing the clinical sensitivity of CF screening.

### 2.3. Assay Validation—Samples

The Archer CF assay was validated using DNA extracted from deidentified DBS. Most samples included in the validation study had already been comprehensively analyzed using the Illumina CSA, Sanger sequencing, qPCR, and/or gap-PCR assays. Validation runs included specimens from unaffected controls, homozygotes, compound heterozygotes, and carriers with representative missense, nonsense, splice site, intronic and intron 9 (8) polyTG/T variants, insertions/deletions (indels), and larger del/dup. Validation samples were batched into six runs of approximately 40 to 80 samples (Table 1). Each run contained a no-template control (NTC) and runs B–F also included a well-characterized Coriell sample (NA12878; NIGMS Human Genetic Cell Repository at the Coriell Institute for Medical Research, Camden, NJ 08103, USA) to monitor assay performance with 50 ng of high-quality DNA.

### 2.4. Assay Validation—DNA Extraction

For NGS, DNA was extracted from 3 mm DBS punches using the QIAamp 96 DNA Blood Kit (Qiagen, Hilden, Germany) per the manufacturer’s instructions. DNA was eluted with 140 µL Tris-HCl, pH 8. Based on the results of the first two validation runs (A and B) with one 3 mm DBS punch per extraction, the decision was made to use two 3 mm DBS punches per extraction. For problematic samples that failed Archer CF quality control (QC) repeatedly, DNA was extracted from up to four 3 mm DBS punches per extraction and successfully genotyped. For Sanger sequencing, fluorescent-based PCR, and qPCR assays, DNA was extracted from one 3 mm DBS punch using a lab-developed method as previously described [10].

### 2.5. Assay Validation—NGS

For the Archer CF assay, library preparation consists of fragmenting genomic DNA; ligating adapters specific for the MiSeq platform, molecular barcodes, and sample indices; and amplifying specific regions of the genome (all 27 *CFTR* exons, intron-exon boundaries, clinically relevant deep intronic and untranslated regions, regions with large del/dup, and the intron 9 (8) polyTG/T region; Table S1). Sequence libraries were prepared per the manufacturer’s recommendations (ArcherDX, Boulder, CO, USA). Validation was performed for libraries prepared manually and for libraries prepared using Sciclone and Zephyr liquid handlers (henceforth, automated; PerkinElmer, Waltham, MA, USA). Libraries were prepared using a 50 μL aliquot of DNA, regardless of concentration. Library yield was assessed using KAPA Library Quantification Kit for Illumina platforms (Roche, Basel, Switzerland) on the Applied Biosystems QuantStudio platform (Thermo Fisher Scientific, Waltham, MA, USA). Following quantification, libraries from each sample were diluted and pooled (16–20 pM for loading). Pooled libraries were denatured and loaded onto standard MiSeq v2 300-cycle flow cells (micro flow cell used for run F only) and run on a MiSeq or MiSeqDx in research use only (RUO) mode (Illumina, San Diego, CA, USA). The KAPA quantification of individual sample libraries has since been replaced with the use of Aline Normalizer Beads (Aline Biosciences, Woburn, MA, USA), which reduces laboratory time.

### 2.6. Assay Validation—Bioinformatics Analyses

Since multiple samples are pooled in the prepared library, raw NGS results include reads for all samples, and the demultiplexing process separates sequence reads for each infant based on sample indices. Base calls and associated quality scores for a NGS run are stored in FASTQ text files. MiSeq Local Run Manager (LRM) or MiSeq Reporter software are used to demultiplex and generate FASTQ files for each sample. The main steps in NGS data analysis are raw read QC, aligning reads to a reference sequence, post-alignment QC, variant calling, and variant interpretation. Following FASTQ file generation, analysis is performed using Archer Analysis v6.0.3.2 or v6.0.4 (ArcherDX). Archer Analysis includes secondary analysis (read trimming/cleaning, de-duplication, error correction, alignment, and variant calling), as well as some tertiary analysis (e.g., annotations and protein effect predictions). Custom scripts were implemented as job hooks in Archer Analysis to be automatically run after standard analyses. Custom report templates were created to summarize various run- and sample-level metrics in pdf format. Custom scripts and report templates are available on github [11].

Default settings in Archer Analysis were used for read trimming/cleaning, de-duplication, error correction, alignment, and variant calling as follows. Alignment was performed using Bowtie 2 [12], against Genome Reference Consortium Human Build 37 (GRCh37/hg19). Two sample-level QC metrics were used to designate individual samples as passing or failing. Sample-level QC metrics required samples to have unique fragment total ≥20,000 and average unique start sites per GSP2 ≥ 30. Initial (default) variant-level QC metrics consisted of alternate allele observation count (AO) ≥ 5, total AO of unique starts (UAO) ≥ 3, and allele fraction (AF) ≥ 0.3. The AF cutoff was lowered to ≥ 0.2 prior to implementation, as described in the results section.

Following alignment, targeted variant calling (second-tier analysis) was performed using a proprietary Archer variant caller, Vision, which uses a targeted-mutation file (TMF) to report genotypes at specific user-defined positions. The second-tier panel used for the validation initially included 294 *CFTR* variants classified as CF-causing by the CFTR2 project (version 31August2018_3) [13], and p.Met607_Gln634del (1949del84), a pathogenic deletion [14] known to be present in NYS CF patients. Following initial validation, but prior to clinical implementation, the TMF was updated to include 338 variants (v1.0.0), including all 334 SNVs and small indels classified as CF-causing by CFTR2 [13] (version 11March2019) and detectable using the Vision caller, and c.350G>A (R117H). R117H is classified as a variant of varying clinical consequence when in cis with the intron 9 (8) 7T allele and CF-causing when in cis with the 5T allele [13], is one of 23 variants recommended for population-based CF carrier screening [15], and is targeted by most commercially available *CFTR* panels. Three additional pathogenic/likely pathogenic variants found in the homozygous state in NYS CF patients were also included c.653T>A (L218X), c.764delT (896delT), and c.935_937delTCT (delF311). The variants included on the current panel (v1.0.0) are shown in Table S2. 

For this validation study, all samples were analyzed using the second-tier targeted DNA panel using the Vision algorithm and third-tier sequence analysis using the FreeBayes SNV/indel algorithm implemented in Archer Analysis [16] using default settings.

Additional third-tier analysis included structural variant (SV) and copy number variant (CNV) calling using proprietary Archer algorithms. Default Archer Analysis SV settings were used, and CNV settings were adjusted to cnv_strong_amplication_threshold=1.3, cnv_strong_deletion_threshold = 0.6, and cnv_p_value_threshold=0.05. The SV algorithm detects structural anomalies in DNA including large deletions, internal tandem duplications, and other structural rearrangements. The custom assay design included primers to target breakpoints of del/dup reported in the CF literature; the SV pipeline utilizes these known breakpoints to target specific del/dup. The CNV algorithm detects copy number gains and losses at the primer level, and does not rely on breakpoint analysis, instead using coverage (i.e., unique reads) relative to a set of normal samples to infer del/dup.

In addition to the three Archer Analysis algorithms, custom scripts were used to assess third-tier region coverage and to determine diplotypes present at the polyTG/T repetitive region of intron 9 (8) that modify the effect of R117H [17] and are classified by CFTR2 as of varying clinical consequence in the absence of R117H [18,19]. The coverage script assesses deduplicated coverage across the targeted region by intersecting mosdepth per-base depth of coverage with the region of interest (ROI) using bedtools and reports the average coverage, uniformity of coverage (defined as % of bases with ≥ average coverage − 20%), and regions/nucleotides with < 10× depth of coverage. The initial script for polyTG/T haplotype determination was inspired by the work of Pagin et al. [20] and used grep to count all reads from a sample’s FASTQ files that fully spanned the polyTG/T region and compared this total to the total number of reads that matched each of the 99 possible haplotypes included in the algorithm [TG(4–14)/T(2–10)]; the ratio of the two most frequent haplotypes detected was used to determine the diplotype for each sample. The polyTG/T script was updated (v1.0.0) prior to clinical implementation to expand haplotypes included in the algorithm to TG(4–14)/T(1–13). After implementation, the polyTG/T script was updated to search the preprocessed BAM files instead of the FASTQ files (v2.0.0). The use of preprocessed BAM files reduced the number of samples that fell into the borderline category.

Variant concordance calculations were performed for inter- and intra-run replicates and for samples with results available from our initial *CFTR* NGS assay (CSA) and other assays. The vcfeval [21] program (Real Time Genomics, Hamilton, New Zealand), was used to compare variants using each sample’s VCF. In practice, Illumina MiSeq Reporter limits the reportable region of the CSA; the complete Illumina VCF files were used for the Illumina to Archer comparison, and therefore, some variants not reported by the CSA were included in the comparison.

### 2.7. Variant Validation

Once implemented for routine NBS, all actionable/reportable variants (see variant interpretation, below) were confirmed using an orthogonal method, including Sanger sequencing, TaqMan assays, gap-PCR, qPCR, or fluorescent-based PCR, depending on variant type [22]. Regions under 10× depth were Sanger sequenced to ensure that all nucleotide positions had sufficient coverage and that variants were not missed due to low coverage. TaqMan assays to validate eight *CFTR* SNVs common in NYS CF cases were implemented, reducing laboratory time and cost compared to Sanger sequencing (Table S3). To confirm del/dup called by NGS, we designed real-time qPCR assays for each of the 27 *CFTR* exons. The qPCR assays were triplex reactions with two *CFTR* targets (except for exon 23 (legacy exon 20); Table S4) and *RPPH1* control reagents (Thermo Fisher Scientific; TaqMan RNase P Assay, ABY dye/QSY probe, product 4485714). Each assay was run in triplicate 10 µL reactions and each run included three calibrator samples, a positive 1-copy heterozygous deletion control (when available), and an NTC. qPCR reactions were performed with standard TaqMan qPCR cycle conditions. qPCR data were analyzed using relative quantitation (2^−ΔΔ*C*t^ method), with the median ΔCt of the calibrators used to calculate the ΔΔCt for each sample and control. Primer and probe sequences used for each qPCR reaction are shown in Table S5. The relative quantity (RQ) range for each copy number was set based on a validated qPCR assay used in our laboratory. RQ ranges were set conservatively with results in an equivocal range requiring repeat testing, shown in Table S6.

### 2.8. Variant Interpretation

Variant classifications were obtained from The Clinical and Functional Translation of CFTR database (CFTR2) [13]. CFTR2 classifies variants as CF-causing, varying clinical consequence, unknown significance, or non-CF-causing. If unknown or not already classified by CFTR2, variants were interpreted according to American College of Medical Genetics and Genomics (ACMG)/Association for Molecular Pathology (AMP) guidelines [23] to classify each detected variant as pathogenic, likely pathogenic, variant of uncertain significance (VOUS), likely benign, or benign. CF-causing, pathogenic, likely pathogenic, varying clinical consequence, and VOUS were considered actionable and included in NBS reports.

## 3. Results

### 3.1. Archer NGS Assay

The Archer CF assay is a comprehensive lab-developed NGS test that allows for simultaneous detection of SNVs/indels, SVs and CNVs. The assay targets all 27 *CFTR* exons, intron-exon boundaries, clinically relevant deep intronic and untranslated regions, large del/dup, and the intron 9 (8) polyTG/T region (Table S1). Second-tier analysis is bioinformatically limited to a defined set of clinically relevant *CFTR* variants. Third-tier analysis is accomplished using three pipelines provided by ArcherDx: (1) SNV and indel detection, (2) SV detection for larger del/dup, and (3) CNV analysis for full or partial gene del/dup, plus (4) a custom script for intron 9 (8) polyTG/T calling.

### 3.2. NGS Validation

Assay accuracy and reproducibility were assessed, following guidelines provided by the NYS Clinical Laboratory Evaluation Program (CLEP) for validation of laboratory-developed tests (LDTs). Including replicates, 273 DNA samples from 227 unique infants were genotyped using the Archer CF assay, passed QC (average unique start sites per GSP2 ≥ 30 and unique fragment total ≥20,000), and were included in the validation analyses. Across all validation runs, the per-sample *total unique reads* were 50,218 ± 30,389 (20,641–286,772; mean ± standard deviation (minimum–maximum)), average unique start sites per gene-specific primer 2 were 87 ± 29 (34–235), and deduplicated coverage was 227 ± 140 (88–1314).

SNV/indel intra-assay reproducibility was assessed by running two samples in duplicate on each run, and inter-assay reproducibility was assessed by running six samples in at least three runs to verify that the same genotypes were obtained. Variants called using both the second-tier TMF panel (Vision algorithm) and via full third-tier SNV/indel analysis (FreeBayes) were compared. The barcodes associated with reproducibility samples differed across each run/replicate to verify that there was no crosstalk between barcodes and that the ability to accurately detect variants was independent of which patient/barcode combinations were used. Intra- and inter-assay reproducibility were both 100% and variants detected included c.224G>A (R75Q), c.350G>A (R117H), c.443T>C (I148T), c.890G>A (R297Q), c.[1210-12T[5]; 1210-34TG[11]] (5T-11TG), c.[1210-12T[5]; 1210-34TG[12]] (5T-12TG), c.1399C>T (L467F), c.1521_1523delCTT (F508del), c.1585-1G>A (1717-1G>A), c.1646G>A (S549N), c.1680-886A>G (1811+1.6kbA>G), c.1820_1903del84 (1949del84), c.2421A>G (I807M), c.2684G>C (S895T), c.3022del (3154delG), c.3041A>G (Y1014C), c.3744delA (3876delA), c.3909C>G (N1303K), and c.3846G>A (W1282X). Non-reportable common variants including c.1408A>G (M470V), c.2562T>G (T854T), c.3870A>G (P1290P), and c.4389G>A (Q1463Q) were also concordant.

Of the 227 infants with samples that passed initial QC, 190 carried one or more of the 338 clinically actionable TMF panel variants. All known second-tier panel variants were called correctly using the Vision algorithm, with 100% sensitivity and 100% specificity, for both the initial (295) and modified (338; v1.0.0) variant panels. Fifty-four different variants, including all but one of the 23 recommended for CF carrier screening (henceforth, ACMG-23) [15] were each detected in at least one sample, and accurately called using the Vision calling algorithm. All samples also underwent complete third-tier sequence analysis using the FreeBayes algorithm; among these 227 infants, 62 additional unique reportable SNV/indel variants were called in the ROI (Table S2).

SNV/indel concordance was calculated using vcfeval for 142 samples that passed QC and had also been tested using the CSA. All variants, including benign and likely benign, present in the ROI reported in Table S2 were used for comparisons; there were an average of four to five variants per sample in the ROI. Clinically actionable variants for the remaining 131 samples screened using other methods that passed QC were manually reviewed and compared. Concordance statistics are shown in Table 1. Initially, there were two false negatives (FNs) in run D. c.2562T>G (2694T/G, T854T) was called but filtered with an AF of 0.235 (default AF filter was 0.3). The sample passed QC, with 54 average unique start sites per GSP2 and 28,530 unique fragments. This variant is not reportable since it is classified as benign. However, the AF filter was subsequently relaxed to 0.2 to minimize the possibility of missed variants. One sample compound heterozygous for c.1519_1521delATC (I507del) and c.1521_1523delCTT (F508del) had both variants correctly called by the Vision algorithm, but c.1521_1523delCTT (F508del) was missed by the FreeBayes algorithm and c.1519_1521delATC (I507del) was called as homozygous, but with an AF of 0.464. The FreeBayes error was reported to the ArcherDx software team, and was attributed to quality filtering of the reads supporting c.1521_1523delCTT (F508del) since these reads had mismatches compared to the reference sequence in the presence of c.1519_1521delATC (I507del). In practice, the results from both algorithms are compared, and confirmation data (Sanger sequence/TaqMan SNP assays) are used to ensure correct genotype(s). There were no false-positive (FP) SNV/indel calls. Considering final genotypes following second- and third-tier analysis, SNV/indel sensitivity was 100% and specificity was 100%.

To test the capability of the Archer CF assay to detect del/dups, data from 198 specimens (155 unique specimens plus replicates and reruns) were analyzed using the Archer SV and CNV analysis pipelines (v6.0.3.2) for del/dup identification. Among the 198, 17 specimens that failed QC for SNV/indel analysis were also considered fails for SV and CNV analyses, leaving 181 specimens from 148 infants for del/dup analysis.

We previously conducted a comprehensive study to assess the spectrum of *CFTR* variants in the NYS CF patient population identified via NBS ([22] and unpublished data). For those studies, variants were genotyped using panels and sequencing, but comprehensive del/dup analysis was not conducted on all samples. Rather, specimens from infants known to have CF but without two clinically relevant *CFTR* variants were screened for del/dup using the Illumina CSA (detects deletions of exons 2–3 and exons 25–26 [22,23]), targeted real-time qPCR assays to assess exon copy number, *CFTR*-specific multiplex ligation-dependent probe amplification (MLPA)), gap-PCR assays, or Sanger sequencing. Twenty-one specimens from seventeen infants with del/dup identified during these studies were included as positive controls in the SV and CNV algorithm validation study. One previously unknown del/dup was identified and validated during this validation study, thus there were a total of 22 true-positive (TP) specimens from 18 infants. The remaining specimens were classified as true negatives (TN) and considered unlikely to carry del/dup, because del/dup are rare, and most of the specimens from infants with CF included in this analysis already had two *CFTR* variants identified.

For the SV analysis, there were 158 TNs, 18 TPs, 4 FNs, 1 FP, and 2 recurrent artifacts (Table S7). Excluding the recurrent artifacts, the SV algorithm’s sensitivity was 81.8% (18/22) and specificity was 99.4% (158/159). For the CNV analysis, there were 153 TNs, 11 TPs, 11 FNs, and 7 FPs. The CNV algorithm’s sensitivity was 50.0% (11/22) and specificity was 95.6% (153/160). Excluding replicate specimens did not change the sensitivity and specificity estimates by more than 4.0% for either algorithm, except for the CNV algorithm’s sensitivity which increased from 50.0% to 61.1%, because six replicates from three individuals with c.1820_1903del (1949del84) that are too small to be detected using the CNV algorithm were tested, contributing to a high FN rate in this group. As shown in Table 2, using a combined approach increased the sensitivity of del/dup detection of either algorithm alone, yielding an overall combined del/dup sensitivity of 100% (22/22) and specificity of 95.0% (152/160). As shown in Table S8, several del/dup were detected by both algorithms. The SV algorithm reliably detected those with known breakpoints, including c.1820_1903del (1949del84) [14] and deletion of exons 2–3 (*CFTR*dele2,3). The CNV algorithm is less robust for detection of smaller del/dup such as a deletion of exons 25–26 (*CFTR*dele22,23), which is only 1534 bp. However, the CNV algorithm increased sensitivity by detecting larger del/dup not specifically targeted by the SV algorithm, including a deletion of exons 1–9 (*CFTR*dele1–8) and duplication of exons 18–21 (*CFTR*dupe16–18). Exon breakpoints reported by the CNV algorithm were not precise, and in several instances, del/dup were shorter or longer than predicted by the software. The CNV algorithm may also be more sensitive to DNA quality and/or quantity, which, in practice, is demonstrated as multiple discontinuous CNV calls in a sample that do not validate. For routine NBS, all del/dup are validated using qPCR, to rule out FP calls prior to reporting.

Specimens from 21 infants were successfully genotyped multiple times, demonstrating inter- and intra-run reproducibility of del/dup detection in the ROI. Del/dup calls from the SV analysis for specimens from two infants heterozygous for c.1820_1903del (1949del84) and 19 specimens with no CNVs were reproducible, using DNA extracted from one or two 3 mm DBS punches, and using the manual and automated library preparation protocols. Del/dup calls from the CNV analysis for the same two specimens heterozygous for c.1820_1903del (1949del84), consistently missed by the CNV algorithm due to small size and 19 specimens with no del/dup were largely reproducible. There were two samples with FP del/dup, each detected in only one of the two to four replicates per infant. All FP del/dups included in the reproducibility study and most from the accuracy study were detected in specimens that used DNA extracted from one 3 mm DBS punch, supporting the use of DNA extracted from two punches.

Of the 227 infants with samples that passed initial QC, there were 203 with known intron 9 (8) polyTG/T haplotypes from the Illumina CSA; results were concordant for 199 infants (98.0%) using the Archer CF assay and the v1.0.0 polyTG/T script and 202 (99.5%) using the v2.0.0 polyTG/T script. The remaining four infants (2.0%) had samples that were borderline with the v1.0.0 polyTG/T script; using the v2.0.0 polyTG/T script, three of four borderline samples were correctly called. In practice, samples with borderline polyTG/T results are reflexed to the confirmatory multiplex PCR polyTG/T assay. The most common polyTG/T haplotype was 7T-11TG, as expected. A range of haplotypes (5,7,8,9)T–(9–13)TG were present in samples included in the validation.

### 3.3. Del/dup qPCR Assay Validation

Eighteen NBS samples from individuals with del/dup in one or more *CFTR* exons were available to validate qPCR assays. Del/dup had been previously identified using the CSA, MLPA, or gap-PCR. Ten additional NBS samples that screened negative for CF were included. All previously identified deletions: c.1820_1903del (1949del84), exons 1–9 (*CFTR*dele1–8), exon 2 (*CFTR*dele2), exons 2–3 (*CFTR*dele2,3), exons 2–4 (*CFTR*dele2–4), exon 12 (*CFTR*dele11), exons 19–21 (*CFTR*dele17a–18), exons 25–26 (*CFTR*dele22,23), and duplications: exons 1–3 (*CFTR*dupe1–3), exons 7–8 (*CFTR*dupe6b,7), exon 22 (*CFTR*dupe19), and exons 25–27 (*CFTR*dupe22–24) were confirmed using the qPCR assays. In addition, a duplication of exons 18–21 (*CFTR*dupe16–18), detected by the Archer assay during this validation was confirmed using qPCR assays. All known del/dup were called using the qPCR assays. After retesting those with equivocal results on the first pass, no FP del/dup were called by the qPCR assays. To establish the reproducibility of the qPCR assays, samples were run in triplicate within runs, in two or three separate runs. All del and dup had RQ values within the expected range with low intra- and inter-run variability.

### 3.4. Prospective Validation Study

Following the establishment of analytical validity, the assay was submitted to and approved by the NYS CLEP for clinical testing. To assess the impact on infrastructure and workflows prior to implementation, a prospective validation study was performed. All NBS samples that were reflexed to the DNA-SEQ portion of the algorithm (Luminex and CSA) in June 2019 were also blinded and reflexed to the Archer CF assay. A total of 1067 NBS samples were tested in 20 batches (representing test days) using the Archer CF assay, in parallel with the routine CF algorithm. Clinically actionable/reportable variants were counted and compared between the assays, and were equivalent for the Archer CF assay and the Luminex and CSA assays.

### 3.5. First Year of Clinical Testing

The custom Archer CF assay was implemented on 1 July 2019, replacing the Luminex assay and CSA. In the first year of clinical testing using the Archer CF assay (through 30 June 2020), more than 220,000 infants were screened for IRT in NYS. Samples from 9557 infants were reflexed to second-tier Archer CF testing. Table 3 shows the results obtained during the first year of screening using the Archer CF assay.

In the first year of screening, 74 of 338 s-tier panel variants have been detected among 9,557 infants with high IRT (Table S9**)**. The five most common panel variants detected were c.1521_1523delCTT (F508del), c.350G>A (R117H), c.3846G>A (W1282X), c.2988+1G>A (3120+1G>A,) and c.1624G>T (G542X). The most common variants (4–7 alleles each) detected that are not included in the ACMG-23 were c.1675G>A (A559T), c.1646G>A (S549N), c.1367T>C (V456A), c.617T>G (L206W), c.1973_1985delGAAATTCAATCCTinsAGAAA (2105-2117del13insAGAAA), and c.2125C>T (R709X).

## 4. Discussion

CF NBS algorithms have had high FP rates in the US and worldwide, leading to the unnecessary referral of unaffected infants, which can lead to anxiety and place a burden on families [24,25]. The high FP rate is primarily due to IRT, the biomarker used for first-tier CF NBS in all states in the US and many other countries worldwide. IRT levels are affected by factors including age at specimen collection, stressful birth, seasonality, and race/ethnicity, and therefore, specificity for CF is low. IRT-DNA algorithms have lower FP rates than IRT-IRT algorithms, and states initially using IRT-IRT algorithms in the US have now implemented IRT-DNA algorithms. However, FP rates using IRT-DNA algorithms are still appreciable because carriers must be referred to maximize sensitivity, especially in regions with diverse populations because affected infants may carry a second rare *CFTR* variant not targeted by variant panels. Furthermore, some states, including NYS, have included an additional failsafe to avoid resulting affected infants with two rare variants as negative by also referring infants with VHIRT and no panel variants [3,26]. Prior to the demonstration that NGS and analysis were feasible and eventually affordable in the NBS/public health lab setting, sequencing a large gene like *CFTR* was impractical.

There are more than 2000 *CFTR* variants cataloged [27], many of which are rare and of uncertain clinical significance. The most common pathogenic *CFTR* variants are SNVs and small indels [15,28] and can be targeted using the Archer Vision algorithm. The Archer CF assay second-tier panel (v1.0.0) currently reports genotypes for 338 clinically relevant *CFTR* variants, including all CF-causing SNVs and small indels in the CFTR2 database (version 11March2019) [13], ACMG-23 [15], and several variants relevant to the NYS population. The previous second-tier panel used by our program targeted 39 variants. The targeted analysis of the Archer CF assay is customizable, allowing the addition or removal of variants from the panel bioinformatically as evidence for *CFTR* variant interpretation accumulates, without assay redesign. The Archer CF assay second-tier panel is modified by adding or removing variants from the TMF, which is a file in VCF format that lists individual variant positions and nucleotide changes to report. The region for which variants are reported in third-tier analysis is also customizable, and modified via editing the chromosome coordinates listed in the “*target_ROI*” field in the GTF file.

Even with the large Archer CF assay second-tier panel, retrospective analysis had previously demonstrated that approximately 8% of alleles in confirmed NYS CF cases were only detected by full bioinformatic analysis, verifying the diverse NYS *CFTR* variant spectrum, and demonstrating the utility of comprehensive third-tier analysis. In addition to full gene sequence analysis, algorithms are used to impute large del/dup typically not detectable by sequencing. Most del/dup are individually rare [29,30], although several recurrent deletions with known breakpoints have been reported in CF patients [31,32,33,34] and have been detected in our population (Table S8). *CFTR* del/dup are not routinely screened due to their rarity, and most are not detected using NGS panels because they are larger than can be consistently called using standard SNV/indel callers. To increase the clinical sensitivity of the custom Archer CF assay, we included primers that target 81 del/dup, based on breakpoints reported in the literature, whereas the CSA targeted two del/dup. Although the combined approach using the Archer SV and CNV pipelines yielded 100% sensitivity in the validation study, it is possible that some del/dup could be missed, especially if small or with untargeted breakpoints. Additional del/dup have since been validated in samples not included in the validation study.

NGS library preparations are more time-consuming than traditional molecular tests. Our sequencing core laboratory prepares Archer CF assay libraries over two partial days, with an overnight reaction, and MiSeq instrument run times are approximately 26 hours. In a lab with staggered shifts, the library preparations could be completed in one day. Compared to the previous IRT-DNA-SEQ algorithm using separate Luminex and CSA assays, the Archer CF assay does increase turnaround time, but only for samples in the IRT top 5% reflexed to the second-tier that have no variants detected; results for infants in this group were typically resulted on day four using the Luminex assay, but most are now typically resulted on day seven using the Archer CF assay, because second-tier analysis now utilizes NGS. However, turnaround times for samples requiring third-tier analysis are similar for the CSA and Archer CF assay. Previously, second- and third-tier testing were run in sequence and required different DNA extraction methods, kits, and assay platforms for the second and third tiers. With the Archer CF assay, DNA from a single batch of samples is extracted, and the DNA is used for both targeted second-tier variant analysis and full third-tier bioinformatic analysis, as necessary. The Archer CF assay also has higher throughput for third-tier analysis, allowing up to 96 samples per run compared to eight for the CSA. The original CSA has recently been replaced by the TruSight Cystic Fibrosis Clinical Sequencing Assay, which now allows 24–96 samples per sequencing run. Although the retest rates due to technical problems during DNA extraction or library preparation are not systematically tracked, the Archer assay had a slightly higher retest rate than the CSA, especially early on, which we attributed at least in part to adapting to protocols and platforms new to the laboratory, as well as liquid handling issues that arose during library preparation, which were not encountered when using manual library preparation for the CSA. In addition, some samples that technically passed default CNV algorithm QC were rerun if CNV plots appeared to have more variability, to assure confidence in the del/dup screening. The variability was likely a result of lower DNA quality in some samples. The higher retest rates have stabilized.

We have made several modifications to methods and bioinformatics. As described, prior to clinical implementation, liquid handling was added to reduce hands-on time required for library preps; bead-based library normalization replaced quantification via qPCR; we upgraded to Archer Analysis 6.0.4. Since implementation, the intron 9 (8) polyTG/T calling algorithm was optimized to reduce borderline calls; a script was implemented to automate second-tier Archer Analysis once a MiSeq run is complete; the GTF file used by Archer Analysis was modified to disable two GSP2s for use by the CNV calling algorithm. These two GSP2s in exon 20 (17b) were associated with FP calls at this exon. The algorithm now utilizes other GSP2s in the region to call del/dup at this exon, and this modification reduced the number of FP calls at this exon. We are currently revalidating a different DNA extraction method that will utilize a single 3 mm punch and reduce the hands-on DNA extraction time.

Our program is exploring multigene NGS panels for other screened conditions. However, implementing sequencing for additional conditions will generate additional variants, including VOUS, requiring evaluation and interpretation by scientists with expertise in clinical genetics. Improvements in variant interpretation are likely as NGS becomes more widespread in the NBS setting. Expansion of expert (CFTR2) or community-based variant curation efforts (ClinVar, ClinGen) can also facilitate this burden.

Implementation of an NGS platform for routine reflex testing in the NBS setting presents opportunities as well as challenges. *CFTR* is a logical first choice for NBS programs to implement sequencing via NGS. First, most NBS programs now have basic molecular capabilities and experience with targeted molecular *CFTR* testing. Next, the genetics of *CFTR* are well established and the CFTR2 project has made highly reliable, expert variant interpretations publicly available [13,35]. Finally, for labs unable to use LDTs or without bioinformatics expertise, the Illumina CSA (now, TruSight) is an accurate and reliable FDA-cleared assay that can be used off-label with DNA extracted from DBS and utilizes a simple and straightforward locked variant calling pipeline. While the infrastructure, including liquid handlers for library preparation and NGS instrumentation, plus computational resources and bioinformatics, can be expensive and complicated, they are expandable and adaptable once established. The development and validation of scripts and custom report templates requires knowledge of programming languages (i.e., bash, python, awk, etc.), however, once scripts and custom report templates are incorporated into the Archer CF assay analysis pipeline, they function via the graphical interface of Archer Analysis for easy, routine clinical use. All assay and analysis specifications, standard operating procedures (SOPs), and scripts are available to the community by request; current scripts used for analysis are posted on github [11].

## Figures and Tables

**Table 1:** Archer CF assay run characteristics and third-tier SNV/indel concordance with CSA.

Run	DBS ^1^	Samples ^2^	Library Prep	MiSeq Reagent Kit	Sensitivity ^3^	Adjusted Sensitivity ^4^	Specificity
A	1	39	M	S	100%	100%	100%
B	1	76	M and A	S	100%	100%	100%
C	2	78	A	S	100%	100%	100%
D	2	78	A	S	98.9%	100%	100%
E	2	38	M	S	100%	100%	100%
F	2	19	M	Mi	100%	100%	100%

M—manual library preparation, A—automated library preparation, S—standard MiSeq reagent kit (300 cycle v2), Mi—micro reagent kit (300 cycle v2). ^1^ DBS—Number dried blood spots used for DNA extraction. ^2^ Includes the total number of DBS samples tested on each run, including QC fails, replicates, and excludes NA12878 and NTCs. Sensitivity and specificity were assessed in the 273 samples that passed QC, and included replicates. ^3^ Sensitivity calculated as number variants correctly detected/total variants across all samples in the ROI for each run. ^4^ Sensitivity after alternative allele fraction (AF) was adjusted to 0.2, as implemented for routine NBS, and both Vision and FreeBayes results were considered.

**Table 2:** Sensitivity and specificity of del/dup calling overall and by the SV and CNV algorithms ^1,2^.

Algorithm	TP	FP	TN	FN	Sensitivity	Specificity
Combined SV and CNV	22	8	152	0	100%	95.0%
Structural variant (SV)	18	1	158	4	81.8%	99.4%
Copy number variant (CNV)	11	7	153	11	50.0%	95.6%

TP—true positive, FP—false positive, TN—true negative, FN—false negative. ^1^ One specimen with a true positive variant (c.1820_1903del (1949del84)) called via the SV algorithm, had 3 FP duplications called via the CNV algorithm; this specimen was considered as both a FP and FN for CNV analysis and overall. ^2^ Del/dup outside of the ROI, including four in introns and one in the 5′UTR are not considered likely to be clinically relevant and were excluded.

**Table 3:** Results from the first year of CF NBS following implementation of Archer CF assay.

Level of Algorithm Reached	Description	Action Required	Number
IRT	Total infants screened	NA	220,878
Second-tier variant panel (TMF)	Top 5% IRT	NA	9557
Third-tier comprehensive CFTR analysis	VHIRT with no variants	Screen negative	165
	One variant	Carrier letter	439
	Two or more variants	Screen positive referral	113

IRT—immunoreactive trypsinogen, NA—not applicable, VHIRT—ultra-high/very high IRT (top 0.1%), TMF—targeted-mutation file. Includes one specimen per infant.

## Supplementary Materials

The following are available online at https://www.mdpi.com/article/10.3390/ijns7040073/s1, Table S1: Region of interest reported by the third-tier Archer CF assay, Table S2: CF Archer second-tier panel content_v1.0, Table S3: *CFTR* TaqMan SNP genotyping assays, Table S4: Multiplex schema for del/dup qPCR assays, Table S5: Del/dup qPCR primer and probe sequences, Table S6: Del/dup qPCR assay relative quantity reference intervals, Table S7: Recurrent artifacts in 58 of 181 specimens analyzed using the SV algorithm, Table S8: Del/dup detected by each algorithm, Table S9: Unique Tier 2 TMF variants identified during the first year of NBS using the Archer CF assay. Supplemental File S1: List of abbreviations and descriptions.
