# Supplementary material for: Validation of a Custom Next-Generation Sequencing Assay for Cystic Fibrosis Newborn Screening
Source: Int J Neonatal Screen. 2021 Nov 2;7(4):73. doi: 10.3390/ijns7040073 (PMC8628990; doi:10.3390/ijns7040073)
Supplement: Supplementary file 1 [file IJNS-07-00073-s001.zip › Sicko et al, NYS CF NGS Panel_Supplemental Tables S1,S3-S9_FINAL-revised.pdf]

**Table S1: Region of interest reported by the third-tier Archer CF assay**

| *CFTR* Exon[1] | Start (hg19) | Stop (hg19) | Reported Region Size |
|---|---|---|---|
| 5'UTR<br>1 | 117,120,041 | 117,120,211 | 171 |
| 2 | 117,144,297 | 117,144,427 | 131 |
| 3 | 117,149,078 | 117,149,206 | 129 |
| 4 | 117,170,943 | 117,171,178 | 236 |
| 5 | 117,174,320 | 117,174,429 | 110 |
| 6 (6a) | 117,175,292 | 117,175,475 | 184 |
| 7 (6b) | 117,176,597 | 117,176,737 | 141 |
| intron 7 (6b) | 117,179,036 | 117,179,049 | 14 |
| 8 (7) | 117,180,144 | 117,180,410 | 267 |
| 9 (8) | 117,182,060 | 117,182,172 | 113 |
| 10 (9) | 117,188,690 | 117,188,887 | 198 |
| 11 (10) | 117,199,508 | 117,199,719 | 212 |
| intron 11 (10) | 117,218,376 | 117,218,386 | 11 |
| 12 (11) | 117,227,783 | 117,227,897 | 115 |
| intron 12 (11) | 117,229,516 | 117,229,535 | 20 |
| 13 (12) | 117,230,397 | 117,230,503 | 107 |
| 14 (13) | 117,231,978 | 117,232,721 | 744 |
| 15 (14a) | 117,234,974 | 117,235,122 | 149 |
| 16 (14b) | 117,242,870 | 117,242,927 | 58 |
| 17 (15) | 117,243,576 | 117,243,846 | 271 |
| 18 (16) | 117,246,718 | 117,246,817 | 100 |
| 19 (17a) | 117,250,563 | 117,250,733 | 171 |
| 20 (17b) | 117,251,605 | 117,251,872 | 268 |
| 21 (18) | 117,254,657 | 117,254,777 | 121 |
| 22 (19) | 117,267,566 | 117,267,834 | 269 |
| intron 22 (19) | 117,267,859 | 117,267,869 | 11 |
| | 117,280,010 | 117,280,020 | 11 |
| 23 (20) | 117,282,482 | 117,282,657 | 176 |
| 24 (21) | 117,292,886 | 117,292,995 | 110 |
| 25 (22) | 117,304,732 | 117,304,924 | 193 |
| 26 (23) | 117,305,503 | 117,305,628 | 126 |
| 27 (24)<br>3'UTR | 117,306,952 | 117,307,262 | 311 |

*UTR* – Untranslated region, *hg19* – human genome assembly GRCh37.

[1] Legacy *CFTR* nomenclature listed in parentheses, if different from HGVS nomenclature.

**Table S3: *CFTR* TaqMan SNP genotyping assays**

| ***CFTR* Variant**[1] | **SNP Genotyping Assay ID**[2] | **dbSNP ID** |
|---|---|---|
| p.Arg117His (R117H) | C__26083773_20 | rs78655421 |
| p.Phe508del (F508del) | C_151693869_10 | rs113993960 |
| p.Gly542* (G542X) | C__11399026_30 | rs113993959 |
| p.Gly551Asp (G551D) | C__32545127_20 | rs75527207 |
| p.Arg553* (R553X) | C__27861430_20 | rs74597325 |
| c.2988+1G>A (3120+1G>A) | C_100964958_20 | rs75096551 |
| p.Asn1303Lys (N1303K) | C__32544994_20 | rs80034486 |
| p.Trp1282* (W1282X) | C__32545014_20 | rs77010898 |

[1]Legacy *CFTR* nomenclature listed in parentheses.

[2] ThermoFisher assay ID.

**Table S4: Multiplex schema for del/dup qPCR assays**

| Assay Name[1] | *CFTR* Exon[1] with FAM probe | *CFTR* Exon[1] with VIC probe |
|---|---|---|
| E1+2 | 2 | 1 |
| E3+4 | 3 | 4 |
| E5+7 (E5+6b) | 7 (6b) | 5 |
| E6+8 (E6a+7) | 8 (7) | 6 (6a) |
| E9+16 (E8+14b) | 9 (8) | 16 (14b) |
| E10+17 (E9+15) | 10 (9) | 17 (15) |
| E11+19 (E10+17a) | 11 (10) | 19 (17a) |
| E12+24 (E11+21) | 12 (11) | 24 (21) |
| E13+22 (E12+19) | 13 (12) | 22 (19) |
| E14+20 (E13+17b) | 14 (13) | 20 (17b) |
| E15+25 (E14a+22) | 15 (14a) | 25 (22) |
| E18+26 (E16+23) | 18 (16) | 26 (23) |
| E21+27 (E18+24) | 21 (18) | 27 (24) |
| E23 (E20) | 23 (20) | N/A |

*del/dup* – deletion/duplication.

All assays multiplexed with *RPPH1* reference assay (ThermoFisher, 4485714) with ABY reporter dye and QSY quencher. FAM probes have an MGB-NFQ quencher, and the VIC probes have a QSY quencher.

[1] Legacy *CFTR* nomenclature listed in parentheses, if different from HGVS nomenclature.

**Table S5: Del/dup qPCR primer and probe sequences**

| ***CFTR* Exon**[1] | **Forward primer sequence (5'>3')** | **Reverse primer sequence (5'>3')** | **Probe Sequence (5'>3')** |
|---|---|---|---|
| 1 | GCAGGCACCCAGAGTAGTAGGT | ACGCCCTCCTCTTTCGTG | TTTGGCATTAGGAGCTTGAGCCCAGA |
| 2 | AGCTGGACCAGACCAATTTTGA | CAGATAGATTGTCAGCAGAATCAACAG | CCTGGAATTGTCAGACATATA |
| 3 | TTTGCACATGCAACTTATTGGTC | TGATACATAATGAATGTACAAATGAGATCC | CTGGCTTCAAAGAAA |
| 4 | AGCCTCTCTTACTGGGAAGAATCATA | GTGATGAAGGCCAAAAATGGC | AGGCTTATGCCTTCTCTTTATTGTGAGGACACTG |
| 5 | TTAAAGCTGTCAAGCCGTGTTCT | GGAAAACTCCGCCTTTCCAG | CAACTTGTTAGTCTCCTTTCCAACAACCTGAACA |
| 6 (6a) | CTTGCATTGGCAYATTTCGT | AATAGGTTGCTACCTGTACTTCATCATC | CAAGTGGCACTCCTCATGGGGCTAATCT |
| 7 (6b) | TCAGAGAGCTGGGAAGATCAGTG | CCATTGCTTCTTCCCAGCAG | TGTGATTACCTCAGAAATGA |
| 8 (7) | GAACTGAAACTGACTCGGAAGGC | AGTGCATAGGGAAGCACAGATAAAA | TGTGAGATACTTCAATAGCTCAG |
| 9 (8) | AATCCTAGTGCTTGGCAAATTAACTTT | AGAAGGCTGTTACATTCTCCATCACTA | CAAGAATATAAGACATTGGAATAT |
| 10 (9) | TGATGACAGCCTCTTCTTCAGTAATT | GAACTACCTTGCCTGCTCCAGT | ATTAATTTCAAGATAGAAAGAGGACAGTT |
| 11 (10) | CTGGAGCCTTCAGAGGGTAAA | TGCTTTGATGAKGCTTCTGTATC | TCCTGGATTATGCCTGGCA |
| 11 (10) | CTGGAGCCTTCAGAGGGTAAA | TGCTTTGATGAKGCTTCTGTATC | TCCTGGATTATGCCTGGCA |
| 12 (11) | ACATCTCCAAGTTTGCAGAGAAAGA | CTTAACCCACTAGCCATAAAACCC | TGAATAACTAATTATTGGTCTAGCAAG |
| 13 (12) | GCATGTAGTGAACTGTTTAAGGCAAAT | CATGAGCATTATAAGTAAGGTATTCAAAGAA | ACACTAGATGACCAGGAAA |
| 14 (13) | AATTTTGCATGAAGGTAGCAGCT | GGTCGAAAGAATCACATCCCA | TCTACAGCCAGACTTTAG |
| 15 (14a) | AATAAAACCACAATGGTGGCATG | ATGTAGTCACTGCTGGTATGCTCTC | AACTGTACTGTCTTATTGTAATAG |
| 16 (14b) | TGTGTACCTTGATATTGGTACACACATC | GAGCCACAGCACAACCAAAGA | ACTGTGTCTTGTTCCATTCCAGGTGGCT |
| 17 (15) | AACAGCTATGCAGTGATTATCACCAG | GACCTCTGAAGAATCCCATAGCA | TATGTGTTTTACATTTACGTGGGAGTAGCCGACA |
| 18 (16) | TGCTAATTCTTATTTGGGTTCTGAATG | GGCAGAAGGTCATCCAAAATTG | CATCTTGTATATTATAGGTGGGATT |
| 19 (17a) | CAGTTGTCGCAGTTTTACAACCC | TTACAAGATGAGTATCGCACATTCAC | TCACAGCAACTCAAACAACTGGAATCTGAAGGTAT |
| 20 (17b) | TCACTCATCTTGTTACAAGCTTAAAAGG | AATGGAAATGAAGGTAACAGCAATG | TTCCACAAAGCTCTGAATTTACATACTGCCAACT |
| 21 (18) | ACATTGCAGTGGGCTGTAAACTC | TCATGCTATTACTCATACTTTGTTACTTGTCT | ATGTGGATAGCTTGGTAAGT |
| 22 (19) | CAAACCATACAAGAATGGCCAAC | AACAAAGCAAGCAGTGTTCAAATC | GCCAAATGACTGTCAAAGATCTCACAGCA |
| 23 (20) | TTTACCTTATAGGTGGGCCTCTTG | TTCTGGCTAAGTCCTTTTGCTCA | AGCCTTTGGAGTGATACC |
| 24 (21) | GAGGTAAGGCTGCTAACTGAAATGA | TGTTGCTCCAGGTATGTTAGGGTAC | AACTCATACCAACACAAATGGCTGATATAGCTGAC |
| 25 (22) | ACATAAGCTTTCAGAACTCCTGTGTTT | CCATCCACAAGGACAAAGTCAA | TTCTCTAACTGCAGGTTGGGCTCAGATCTG |
| 26 (23) | AAGCATTTGCTGATTGCACAGT | AAGGGCAATGAGATCTTAAGTAAAGTTATAA | AGGATAGAAGCAATGCTGGAATGCCAACA |
| 27 (24) | AGGCTCTGGACATTGCATTCTT | TGCTTGAGTTCCGGTGGG | TCCTTTGAGCCTGTGCCAGTTTCTGTC |

*del/dup* – deletion/duplication.

[1] Legacy *CFTR* nomenclature listed in parentheses, if different from HGVS nomenclature.

**Table S6: Del/dup qPCR assay relative quantity reference intervals**

| Average RQ | Copy Number | Interpretation |
|---|---|---|
| Undetermined | 0 | Homozygous for *CFTR* exon deletion |
| 0.001 – 0.299 | Unknown | Equivocal results |
| 0.300 – 0.599 | 1 | Heterozygous for *CFTR* exon deletion |
| 0.600 – 0.799 | Unknown | Equivocal results |
| 0.800 – 1.200 | 2 | Normal |
| 1.201 – 1.249 | Unknown | Equivocal results |
| ≥1.250 | ≥3 | Amplification[1] |

*RQ* – relative quantity.

[1] Positive deletion and duplication controls were not available for all exons. qPCR assays were validated to detect heterozygous deletions including exons: 1-9,12,14,19-21 and 25-26 (1-8,11,13,17a-18 and 22-23), and duplications/amplifications including exons: 1-3,7-8,18-22 and 25-27 (1-3,6b-7,16-19 and 22-24).

**Table S7: Recurrent artifacts in 58 of 181 specimens analyzed using the SV algorithm**

| *CFTR* Exon(s) Affected [1] | Del/dup | Number Start Sites[2] | Number Reads[2] | % Reads[2] | Strong SV[2] | Breakpoint (hg19)[2] | Breakpoint (hg19)[2] | Size (bp)[3] |
|---|---|---|---|---|---|---|---|---|
| intron 20 (intron 17b) | Del | 5 – 126 | 6 – 457 | 2.1 – 92.6 | Y | Variable. minimum observed: 117,252,060 | Variable. maximum observed: 117,252,122 | 46 – 52 |
| 27 - 3'UTR (24 - 3'UTR) | Del | 4 – 19 | 6 – 35 | 0.5 – 12.1 | Y | 117,307,228 | 117,307,260 | 33 |
| 27 - 3'UTR (24 - 3'UTR) | Del | 4 – 14 | 5 – 29 | 0.7 – 8.3 | Y | 117,307,194 | 117,307,226 | 33 |

*Del* – deletion, *Dup* – duplication, *hg19* – human genome assembly GRCh37, *bp* – base pair, *UTR* – untranslated region.

[1]Legacy exon *CFTR* nomenclature listed in parentheses. [2] Output from SV algorithm. [3] Size determined using the SV algorithm.

**Table S8: Del/dup detected by each algorithm**

| *CFTR* Exon(s) Affected | Del/ Dup | HGVS Nomenclature[1,4] | # NYS alleles tested[3] | Vision/ FreeBayes[2,3] | SV[3,4] | CNV[3,4] | Comments |
|---|---|---|---|---|---|---|---|
| 14 (13) | Del | c.1820_1903del | 12 | 12/12 | 12/12 | 0/12 | Legacy=1949del84. Too small to be detected by CNV algorithm. |
| 1-9 (1-8) | Del | c.(?_1)_( 1209+1_1210-1)del | 1 | 0/1 | 0/1 | 1/1 | Breakpoints not included in assay design. |
| 2 | Del | c.(53+1_54-1)_(164+1_165-1)del | 2 | 0/2 | 0/2 | 2/2 | Breakpoints for several reported deletions in exon 2 were targeted; but the exact exon 2 breakpoints in these samples (unknown) may not have been included. |
| 2-3 | Del | c.54-5940_273+10250del | 3 | 0/3 | 3/3 | 3/3 | Called using SV and CNV algorithms. |
| 2-4 | Del | c.(53+1_54-1)_(489+1_490-1)del | 1 | 0/1 | 1/1 | 1/1 | Called using SV and CNV algorithms. |
| 18-21 (16-18) | Dup | c.(2908+1_2909-1)_(3468+1_3469-1)dup | 1 | 0/1 | 0/1 | 1/1 | Breakpoints not included in assay design. |
| 19-21 (17a-18) | Del | c.2988+1168_3468+2108del | 2 | 0/2 | 2/2 | 2/2 | Reliably called using SV and CNV algorithms. |
| 25-26 (22-23) | Del | c.3964-82_4242+575del | 2 | 0/2 | 2/2 | 1/2 | Likely too small to be reliably detected by CNV algorithm. |

*Del* – deletion, *Dup* – duplication, *SV* – structural variant algorithm, *CNV* – copy number variant algorithm.

[1] HGVS nomenclature relative to NC_000007.13 and NM_000492.3.

[2] Vision and FreeBayes both consistently detected c.1820_1903del (1949del84).

[3] Number detected / number tested using each algorithm. Counts include replicate samples.

[4] Exact breakpoints were unknown for most del/dup, except where specified. Breakpoints reported by the Archer Analysis SV algorithm were slightly different than reported in literature for some del/dup. For example, the deletion at exons 25-26 is reported in CFTR2 as c.3964-78_4242+577del.

**Table S9: Unique Tier 2 TMF variants identified during the first year of NBS using the Archer CF assay**

| Variant Frequency | Protein (NP_000483.3) | cDNA (NM_000492.3) | Legacy Name |
|---|---|---|---|
| Common | p.Phe508del | c.1521_1523delCTT[1,2] | F508del |
| Recurrent (≥5 alleles) | p.Arg117His | c.350G>A[1,2] | R117H |
| | p.Trp1282* | c.3846G>A[1,2] | W1282X |
| | N/A | c.2988+1G>A[1,2] | 3120+1G>A |
| | p.Gly542* | c.1624G>T[1,2] | G542X |
| | N/A | c.3718-2477C>T[1,2] | 3849+10kbC>T |
| | p.Gly551Asp | c.1652G>A[1,2] | G551D |
| | N/A | c.2657+5G>A[1,2] | 2789+5G>A |
| | p.Ala559Thr | c.1675G>A[2] | A559T |
| | p.Asn1303Lys | c.3909C>G[1,2] | N1303K |
| | p.Ser549Asn | c.1646G>A[2] | S549N |
| | p.Val456Ala | c.1367T>C | V456A |
| | p.Leu206Trp | c.617T>G | L206W |
| | p.Arg334Trp | c.1000C>T[1,2] | R334W |
| | p.Arg553* | c.1657C>T[1,2] | R553X |
| Rare (2-4 alleles) | p.Arg658Lysfs*4 | c.1973_1985delGAAATTCAATCCTinsAGAAA | 2105-2117del13insAGAAA |
| | p.Ile507del | c.1519_1521delATC[1,2] | I507del |
| | p.Arg1162* | c.3484C>T[1,2] | R1162X |
| | p.Arg709* | c.2125C>T | R709X |
| | N/A | c.1585-1G>A[1,2] | 1717-1G>A |
| | p.Gln685Thrfs*4 | c.2052dupA | 2184insA |
| | N/A | c.489+1G>T[1,2] | 621+1G>T |
| | p.Gly85Glu | c.254G>A[1,2] | G85E |
| | p.Leu15Pro | c.44T>C | L15P |
| | p.Arg1158* | c.3472C>T | R1158X |
| | p.Arg117Cys | c.349C>T | R117C |
| | p.Arg560Thr | c.1679G>C[1,2] | R560T |
| | p.Glu726Argfs*4 | c.2175dupA[2] | 2307insA |
| | N/A | c.3140-26A>G | 3272-26A>G |
| | p.Lys1250Argfs*9 | c.3744delA[2] | 3876delA |
| | N/A | c.579+3A>G | 711+3A>G |
| | p.Phe312del | c.935_937delTCT | F312del or F311del |
| | p.Leu1254* | c.3761T>G | L1254X |
| | p.Arg347Pro | c.1040G>C[1,2] | R347P |
| | p.Arg352Gln | c.1055G>A | R352Q |
| | p.Thr1036Asn | c.3107C>A | T1036N |
| Private (1 allele) | p.Cys343* | c.1029delC | 1161delC |
| | N/A | c.1209+1G>A | 1341+1G>A |
| | p.Ile444Argfs*3 | c.1327_1330dupGATA | 1461ins4 |
| | p.Lys447Argfs*2 | c.1340delA | 1471delA |
| | p.Gly473Glufs*54 | c.1418delG | 1548delG |
| | N/A | c.1680-877G>T | 1811+1643G>T |
| | N/A | c.1766+1G>A[1,2] | 1898+1G>A |
| | p.Lys684Serfs*38 | c.2051_2052delAAinsG[2,3] | 2183AA>G |
| | N/A | c.2490+1G>A | 2622+1G>A |
| | N/A | c.164+1G>T | 296+1G>T |
| | N/A | c.165-3C>T | 297-3C>T |
| | p.Thr1220Lysfs*8 | c.3659delC | 3791delC |
| | p.Trp79Leufs*32 | c.233dupT | 365-366insT |
| | p.Leu1258Phefs*7 | c.3773dupT[2] | 3905insT |
| | p.Ile1295Phefs*32 | c.3883_3886delATTT | 4010del4 |
| | p.Glu1418Argfs*14 | c.4251delA | 4382delA |

| Variant Frequency | Protein (NP_000483.3) | cDNA (NM_000492.3) | Legacy Name |
|---|---|---|---|
| | N/A | c.579+1G>T[1,2] | 711+1G>T |
| | p.Asn268Ilefs*17 | c.803delA | 935delA |
| | p.Asn287Lysfs*19 | c.861_865delCTTAA | 991del5 |
| | p.Ala455Glu | c.1364C>A[1,2] | A455E |
| | p.Ala613Thr | c.1837G>A | A613T |
| | p.Cys276* | c.828C>A | C276X |
| | p.Asp110His | c.328G>C | D110H |
| | p.Glu60* | c.178G>T | E60X |
| | p.Gly1244Glu | c.3731G>A | G1244E |
| | p.Lys710* | c.2128A>T | K710X |
| | p.Pro67Leu | c.200C>T | P67L |
| | p.Gln98* | c.292C>T | Q98X |
| | p.Gln996= | c.2988G>A | 3120G>A or Q996Q |
| | p.Arg1066Cys | c.3196C>T | R1066C |
| | p.Arg75* | c.223C>T | R75X |
| | p.Ser1159Phe | c.3476C>T | S1159F |
| | p.Ser1255Pro | c.3763T>C | S1255P |
| | p.Ser466* | c.1397C>G | S466X(TAG) |
| | p.Val232Asp | c.695T>A | V232D |
| | p.Trp1098* | c.3293G>A | W1098X(TAG) |
| | p.Trp57* | c.171G>A | W57X |
| | p.Tyr1092* | c.3276C>A | Y1092X |

N/A – Not applicable. Variants are ordered by frequency, including carriers and infants referred.

[1] ACMG-23 variant.

[2] Luminex xTAG CF39v2 variant.

[3] Vision variant caller reports both c.2052delA and c.2051_2052delAAinsG for samples that are heterozygous for c.2051_2052delAAinsG; FreeBayes variant caller and Sanger sequencing confirm the correct call is only c.2051_2052delAAinsG.
