# Supplementary material for: Validation of a Custom Next-Generation Sequencing Assay for Cystic Fibrosis Newborn Screening
Source: Int J Neonatal Screen. 2021 Nov 2;7(4):73. doi: 10.3390/ijns7040073 (PMC8628990; doi:10.3390/ijns7040073)
Supplement: Supplementary file 1 [file IJNS-07-00073-s001.zip › Sicko et al, NYS CF NGS Panel_Supplemental_Table S2.pdf]

NYS Cystic Fibrosis Newborn Screening: Custom *CFTR* Variant Panel Content

<table>
<tr><th>Variant: Legacy | HGVS Protein (cDNA)</th></tr>
<tr><td>124del23bp | p.? (c.-9_14delCGA
GAGACCATGCAGAGGTCGCC)</td></tr>
<tr><td>M1V | p.Met1Val / p.Met1? (c.1A>G)</td></tr>
<tr><td>Q2X | p.Gln2* (c.4C>T)</td></tr>
<tr><td>S4X | p.Ser4* (c.11C>A)</td></tr>
<tr><td>S13F | p.Ser13Phe (c.38C>T)</td></tr>
<tr><td>182delT | p.Phe17Serfs*8 (c.50delT)</td></tr>
<tr><td>L15P | p.Leu15Pro (c.44T>C)</td></tr>
<tr><td>185+1G->T | p.? (c.53+1G>T)</td></tr>
<tr><td>W19X | p.Trp19* (c.57G>A)</td></tr>
<tr><td>G27R | p.Gly27Arg (c.79G>A)</td></tr>
<tr><td>G27X | p.Gly27* (c.79G>T)</td></tr>
<tr><td>Q30X | p.Gln30* (c.88C>T)</td></tr>
<tr><td>Q39X | p.Gln39* (c.115C>T)</td></tr>
<tr><td>A46D | p.Ala46Asp (c.137C>A)</td></tr>
<tr><td>296+1G->A | p.? (c.164+1G>A)</td></tr>
<tr><td>296+1G->T | p.? (c.164+1G>T)</td></tr>
<tr><td>296+2T->C | p.? (c.164+2T>C)</td></tr>
<tr><td>296+3insT | p.? (c.164+4dupT)</td></tr>
<tr><td>297-3C->T | p.? (c.165-3C>T)</td></tr>
<tr><td>297-1G->A | p.? (c.165-1G>A)</td></tr>
<tr><td>E56K | p.Glu56Lys (c.166G>A)</td></tr>
<tr><td>W57G | p.Trp57Gly (c.169T>G)</td></tr>
<tr><td>W57X | p.Trp57* (c.170G>A)</td></tr>
<tr><td>W57X | p.Trp57* (c.171G>A)</td></tr>
<tr><td>306delTAGA | p.Asp58Glufs*32
(c.174_177delTAGA)</td></tr>
<tr><td>306insA | p.Arg59Lysfs*10 (c.175dupA)</td></tr>
<tr><td>E60K | p.Glu60Lys (c.178G>A)</td></tr>
<tr><td>E60X | p.Glu60* (c.178G>T)</td></tr>
<tr><td>P67L | p.Pro67Leu (c.200C>T)</td></tr>
<tr><td>R75X | p.Arg75* (c.223C>T)</td></tr>
<tr><td>365-366insT | p.Trp79Leufs*32 (c.233dupT)</td></tr>
<tr><td>G85E | p.Gly85Glu (c.254G>A)</td></tr>
<tr><td>394delTT | p.Leu88Ilefs*22
(c.262_263delTT)</td></tr>
<tr><td>L88X | p.Leu88* (c.263T>A)</td></tr>
<tr><td>L88X | p.Leu88* (c.263T>G)</td></tr>
<tr><td>G91R | p.Gly91Arg (c.271G>A)</td></tr>
<tr><td>405+1G->A | p.? (c.273+1G>A)</td></tr>
<tr><td>405+3A->C | p.? (c.273+3A>C)</td></tr>
<tr><td>406-2A->G | p.? (c.274-2A>G)</td></tr>
<tr><td>406-1G->A | p.? (c.274-1G>A)</td></tr>
<tr><td>E92K | p.Glu92Lys (c.274G>A)</td></tr>
<tr><td>E92X | p.Glu92* (c.274G>T)</td></tr>
<tr><td>Q98X | p.Gln98* (c.292C>T)</td></tr>
<tr><td>Q98R | p.Gln98Arg (c.293A>G)</td></tr>
<tr><td>P99L | p.Pro99Leu (c.296C>T)</td></tr>
<tr><td>L102R | p.Leu102Arg (c.305T>G)</td></tr>
<tr><td>442delA | p.Arg104Glufs*3 (c.310delA)</td></tr>
<tr><td>444delA | p.Ile105Serfs*2 (c.313delA)</td></tr>
<tr><td>457TAT->G | p.Tyr109Glyfs*4
(c.325_327delTATinsG)</td></tr>
<tr><td>D110H | p.Asp110His (c.328G>C)</td></tr>
<tr><td>R117C | p.Arg117Cys (c.349C>T)</td></tr>
<tr><td>R117H | p.Arg117His (c.350G>A)[ʃ]</td></tr>
<tr><td>Y122X | p.Tyr122* (c.366T>A)</td></tr>
<tr><td>G126D | p.Gly126Asp (c.377G>A)</td></tr>
<tr><td>541delC | p.Leu137Serfs*16 (c.409delC)</td></tr>
<tr><td>L138ins | p.Leu138dup
(c.413_415dupTAC)</td></tr>
<tr><td>H139R | p.His139Arg (c.416A>G)</td></tr>
<tr><td>574delA | p.Ile148Leufs*5 (c.442delA)</td></tr>
<tr><td>602del14 | p.Phe157*
(c.470_483delTTAGTTTGATTTAT)</td></tr>
<tr><td>Y161D | p.Tyr161Asp (c.481T>G)</td></tr>
<tr><td>621+1G->T | p.? (c.489+1G>T)</td></tr>
<tr><td>L165S | p.Leu165Ser (c.494T>C)</td></tr>
<tr><td>663delT | p.Ile177Metfs*12 (c.531delT)</td></tr>
<tr><td>G178R | p.Gly178Arg (c.532G>A)</td></tr>
<tr><td>675del4 | p.Leu183Phefs*5
(c.543_546delTAGT)</td></tr>
<tr><td>E193X | p.Glu193* (c.577G>T)</td></tr>
<tr><td>711+1G->T | p.? (c.579+1G>T)</td></tr>
<tr><td>711+3A->G | p.? (c.579+3A>G)</td></tr>
<tr><td>711+5G->A | p.? (c.579+5G>A)</td></tr>
<tr><td>712-1G->T | p.? (c.580-1G>T)</td></tr>
</table>

<table>
<tr><th>Variant: Legacy | HGVS Protein (cDNA)</th></tr>
<tr><td>H199Y | p.His199Tyr (c.595C>T)</td></tr>
<tr><td>P205S | p.Pro205Ser (c.613C>T)</td></tr>
<tr><td>L206W | p.Leu206Trp (c.617T>G)</td></tr>
<tr><td>W216X | p.Trp216* (c.647G>A)</td></tr>
<tr><td>L218X | p.Leu218* (c.653T>A)[+]</td></tr>
<tr><td>Q220X | p.Gln220* (c.658C>T)</td></tr>
<tr><td>L227R | p.Leu227Arg (c.680T>G)</td></tr>
<tr><td>V232D | p.Val232Asp (c.695T>A)</td></tr>
<tr><td>849delG | p.Leu240* (c.717delG)</td></tr>
<tr><td>852del22 | p.Gly241Glufs*13 (c.723_
743+1delGAGAATGATGATGAAGTACAGG)</td></tr>
<tr><td>896delT | p.Ile255Thrfs*6 (c.764delT)[+]</td></tr>
<tr><td>935delA | p.Asn268Ilefs*17 (c.803delA)</td></tr>
<tr><td>Y275X | p.Tyr275* (c.825C>G)</td></tr>
<tr><td>C276X | p.Cys276* (c.828C>A)</td></tr>
<tr><td>977insA | p.Met284Asnfs*3 (c.850dupA)</td></tr>
<tr><td>991del5 | p.Asn287Lysfs*19
(c.861_865delCTTAA)</td></tr>
<tr><td>delF311 | p.Phe312del (c.935_937delTCT)[§]</td></tr>
<tr><td>F311L | p.Phe311Leu (c.933C>G)</td></tr>
<tr><td>1078delT | p.Phe316Leufs*12 (c.948delT)</td></tr>
<tr><td>1119delA | p.Gly330Glufs*39 (c.987delA)</td></tr>
<tr><td>G330X | p.Gly330* (c.988G>T)</td></tr>
<tr><td>R334W | p.Arg334Trp (c.1000C>T)</td></tr>
<tr><td>R334L | p.Arg334Leu (c.1001G>T)</td></tr>
<tr><td>1138insG | p.Ile336Serfs*28
(c.1006_1007insG)</td></tr>
<tr><td>I336K | p.Ile336Lys (c.1007T>A)</td></tr>
<tr><td>T338I | p.Thr338Ile (c.1013C>T)</td></tr>
<tr><td>1154insTC | p.Phe342Hisfs*28
(c.1021_1022dupTC)</td></tr>
<tr><td>S341P | p.Ser341Pro (c.1021T>C)</td></tr>
<tr><td>1161delC | p.Cys343* (c.1029delC)</td></tr>
<tr><td>L346P | p.Leu346Pro (c.1037T>C)</td></tr>
<tr><td>R347H | p.Arg347His (c.1040G>A)</td></tr>
<tr><td>R347P | p.Arg347Pro (c.1040G>C)</td></tr>
<tr><td>R352Q | p.Arg352Gln (c.1055G>A)</td></tr>
<tr><td>Q359K/T360K | p.Gln359_Thr360delins
LysLys (c.1075_1079delCAAACinsAAAAA)</td></tr>
<tr><td>1213delT | p.Trp361Glyfs*8 (c.1081delT)</td></tr>
<tr><td>1248+1G->A | p.? (c.1116+1G>A)</td></tr>
<tr><td>1249-1G->A | p.? (c.1117-1G>A)</td></tr>
<tr><td>1259insA | p.Gln378Alafs*4 (c.1130dupA)</td></tr>
<tr><td>1288insTA | p.Asn386Ilefs*3
(c.1155_1156dupTA)</td></tr>
<tr><td>W401X | p.Trp401* (c.1202G>A)</td></tr>
<tr><td>W401X | p.Trp401* (c.1203G>A)</td></tr>
<tr><td>1341+1G->A | p.? (c.1209+1G>A)</td></tr>
<tr><td>1343delG | p.Gly404Aspfs*38 (c.1211delG)</td></tr>
<tr><td>Q414X | p.Gln414* (c.1240C>T)</td></tr>
<tr><td>1429del7 | p.Ser434Leufs*6
(c.1301_1307delCACTTCT)</td></tr>
<tr><td>1461ins4 | p.Ile444Argfs*3
(c.1327_1330dupGATA)</td></tr>
<tr><td>1471delA | p.Lys447Argfs*2 (c.1340delA)</td></tr>
<tr><td>L453S | p.Leu453Ser (c.1358T>C)</td></tr>
<tr><td>A455E | p.Ala455Glu (c.1364C>A)</td></tr>
<tr><td>1497delGG | p.Val456Cysfs*25
(c.1365_1366delGG)</td></tr>
<tr><td>V456A | p.Val456Ala (c.1367T>C)</td></tr>
<tr><td>1504delG | p.Gly458Aspfs*11 (c.1373delG)</td></tr>
<tr><td>1525-2A->G | p.? (c.1393-2A>G)</td></tr>
<tr><td>1525-1G->A | p.? (c.1393-1G>A)</td></tr>
<tr><td>S466X | p.Ser466* (c.1397C>A)</td></tr>
<tr><td>S466X | p.Ser466* (c.1397C>G)</td></tr>
<tr><td>L467P | p.Leu467Pro (c.1400T>C)</td></tr>
<tr><td>1548delG | p.Gly473Glufs*54 (c.1418delG)</td></tr>
<tr><td>E474K | p.Glu474Lys (c.1420G>A)</td></tr>
<tr><td>S489X | p.Ser489* (c.1466C>A)</td></tr>
<tr><td>S492F | p.Ser492Phe (c.1475C>T)</td></tr>
<tr><td>1609delCA | p.Gln493Valfs*10
(c.1477_1478delCA)</td></tr>
<tr><td>Q493X | p.Gln493* (c.1477C>T)</td></tr>
<tr><td>W496X | p.Trp496* (c.1487G>A)</td></tr>
<tr><td>I502T | p.Ile502Thr (c.1505T>C)</td></tr>
<tr><td>I507del | p.Ile507del
(c.1519_1521delATC)</td></tr>
</table>

<table>
<tr><th>Variant: Legacy | HGVS Protein (cDNA)</th></tr>
<tr><td>F508del | p.Phe508del
(c.1521_1523delCTT)</td></tr>
<tr><td>D513G | p.Asp513Gly (c.1538A>G)</td></tr>
<tr><td>1677delTA | p.Tyr515* (c.1545_1546delTA)</td></tr>
<tr><td>V520F | p.Val520Phe (c.1558G>T)</td></tr>
<tr><td>C524X | p.Cys524* (c.1572C>A)</td></tr>
<tr><td>Q525X | p.Gln525* (c.1573C>T)</td></tr>
<tr><td>1716+1G->A | p.? (c.1584+1G>A)</td></tr>
<tr><td>1717-8G->A | p.? (c.1585-8G>A)</td></tr>
<tr><td>1717-1G->A | p.? (c.1585-1G>A)</td></tr>
<tr><td>G542X | p.Gly542* (c.1624G>T)</td></tr>
<tr><td>S549R | p.Ser549Arg (c.1645A>C)</td></tr>
<tr><td>S549N | p.Ser549Asn (c.1646G>A)</td></tr>
<tr><td>S549R | p.Ser549Arg (c.1647T>A)</td></tr>
<tr><td>S549R | p.Ser549Arg (c.1647T>G)</td></tr>
<tr><td>G550X | p.Gly550* (c.1648G>T)</td></tr>
<tr><td>1782delA | p.Gly551Valfs*8 (c.1650delA)</td></tr>
<tr><td>G551S | p.Gly551Ser (c.1651G>A)</td></tr>
<tr><td>G551D | p.Gly551Asp (c.1652G>A)</td></tr>
<tr><td>Q552X | p.Gln552* (c.1654C>T)</td></tr>
<tr><td>R553X | p.Arg553* (c.1657C>T)</td></tr>
<tr><td>1802delC | p.Ser557Phefs*2 (c.1670delC)</td></tr>
<tr><td>L558S | p.Leu558Ser (c.1673T>C)</td></tr>
<tr><td>A559T | p.Ala559Thr (c.1675G>A)</td></tr>
<tr><td>R560K | p.Arg560Lys (c.1679G>A)</td></tr>
<tr><td>R560T | p.Arg560Thr (c.1679G>C)</td></tr>
<tr><td>1811+1G->A | p.? (c.1679+1G>A)</td></tr>
<tr><td>1811+1G->C | p.? (c.1679+1G>C)</td></tr>
<tr><td>1811+1.6kbA->G | p.? (c.1680-886A>G)</td></tr>
<tr><td>1811+1643G->T | p.? (c.1680-877G>T)</td></tr>
<tr><td>1812-1G->A | p.? (c.1680-1G>A)</td></tr>
<tr><td>R560S | p.Arg560Ser (c.1680A>C)</td></tr>
<tr><td>A561E | p.Ala561Glu (c.1682C>A)</td></tr>
<tr><td>Y563N | p.Tyr563Asn (c.1687T>A)</td></tr>
<tr><td>Y563D | p.Tyr563Asp (c.1687T>G)</td></tr>
<tr><td>1824delA | p.Asp565Metfs*7 (c.1692delA)</td></tr>
<tr><td>1833delT | p.Leu568Cysfs*4 (c.1703delT)</td></tr>
<tr><td>Y569D | p.Tyr569Asp (c.1705T>G)</td></tr>
<tr><td>P574H | p.Pro574His (c.1721C>A)</td></tr>
<tr><td>E585X | p.Glu585* (c.1753G>T)</td></tr>
<tr><td>1898+1G->A | p.? (c.1766+1G>A)</td></tr>
<tr><td>1898+1G->C | p.? (c.1766+1G>C)</td></tr>
<tr><td>1898+1G->T | p.? (c.1766+1G>T)</td></tr>
<tr><td>1898+3A->G | p.? (c.1766+3A>G)</td></tr>
<tr><td>1898+5G->T | p.? (c.1766+5G>T)</td></tr>
<tr><td>1924del7 | p.Lys598Glyfs*11
(c.1792_1798delAAAACTA)</td></tr>
<tr><td>1949del84 | p.Met607_Gln634del
(c.1820_1903del84)[+]</td></tr>
<tr><td>H609R | p.His609Arg (c.1826A>G)</td></tr>
<tr><td>A613T | p.Ala613Thr (c.1837G>A)</td></tr>
<tr><td>G628R | p.Gly628Arg (c.1882G>A)</td></tr>
<tr><td>G628R | p.Gly628Arg (c.1882G>C)</td></tr>
<tr><td>2055del9->A | p.Ser641Argfs*5
(c.1923_1931delCTCAAAACTinsA)</td></tr>
<tr><td>2075delA | p.Asp648Valfs*15 (c.1943delA)</td></tr>
<tr><td>2105-2117del13insAGAAA |
p.Arg658Lysfs*4 (c.1973_1985delGA
AATTCAATCCTinsAGAAA)</td></tr>
<tr><td>2118del4 | p.Thr663Argfs*8
(c.1986_1989delAACT)</td></tr>
<tr><td>2143delT | p.Leu671* (c.2012delT)</td></tr>
<tr><td>G673X | p.Gly673* (c.2017G>T)</td></tr>
<tr><td>2184delA | p.Lys684Asnfs*38
(c.2052delA)</td></tr>
<tr><td>2184insA | p.Gln685Thrfs*4 (c.2052dupA)</td></tr>
<tr><td>2183AA->G | p.Lys684Serfs*38
(c.2051_2052delAAinsG)</td></tr>
<tr><td>2185insC | p.Gln685Profs*4 (c.2053dupC)</td></tr>
<tr><td>Q685X | p.Gln685* (c.2053C>T)</td></tr>
<tr><td>R709X | p.Arg709* (c.2125C>T)</td></tr>
<tr><td>K710X | p.Lys710* (c.2128A>T)</td></tr>
<tr><td>Q715X | p.Gln715* (c.2143C>T)</td></tr>
<tr><td>Q720X | p.Gln720* (c.2158C>T)</td></tr>
<tr><td>2307insA | p.Glu726Argfs*4 (c.2175dupA)</td></tr>
<tr><td>L732X | p.Leu732* (c.2195T>G)</td></tr>
<tr><td>2347delG | p.Val739Tyrfs*16 (c.2215delG)</td></tr>
</table>

<table>
<tr><th>Variant: Legacy | HGVS Protein (cDNA)</th></tr>
<tr><td>2372del8 | p.Ile748Serfs*28
(c.2241_2248delGATACTGC)</td></tr>
<tr><td>R764X | p.Arg764* (c.2290C>T)</td></tr>
<tr><td>R785X | p.Arg785* (c.2353C>T)</td></tr>
<tr><td>R792X | p.Arg792* (c.2374C>T)</td></tr>
<tr><td>2556insAT | p.Ser809Ilefs*13
(c.2423_2424dupAT)</td></tr>
<tr><td>2585delT | p.Leu818Trpfs*3 (c.2453delT)</td></tr>
<tr><td>2594delGT | p.Ser821Argfs*4
(c.2463_2464delTG)</td></tr>
<tr><td>E822X | p.Glu822* (c.2464G>T)</td></tr>
<tr><td>2622+1G->A | p.? (c.2490+1G>A)</td></tr>
<tr><td>E831X | p.Glu831* (c.2491G>T)</td></tr>
<tr><td>W846X | p.Trp846* (c.2537G>A)</td></tr>
<tr><td>W846X | p.Trp846* (c.2538G>A)</td></tr>
<tr><td>Y849X | p.Tyr849* (c.2547C>A)</td></tr>
<tr><td>R851X | p.Arg851* (c.2551C>T)</td></tr>
<tr><td>2711delT | p.Phe861Leufs*3 (c.2583delT)</td></tr>
<tr><td>2721del11 | p.Ile864Serfs*28
(c.2589_2599delAATTTGGTGCT)</td></tr>
<tr><td>2732insA | p.Val868Serfs*28 (c.2601dupA)</td></tr>
<tr><td>W882X | p.Trp882* (c.2645G>A)</td></tr>
<tr><td>2789+5G->A | p.? (c.2657+5G>A)</td></tr>
<tr><td>2790-1G->C | p.? (c.2658-1G>C)</td></tr>
<tr><td>Q890X | p.Gln890* (c.2668C>T)</td></tr>
<tr><td>S912X | p.Ser912* (c.2735C>A)</td></tr>
<tr><td>2869insG | p.Tyr913* (c.2737_2738insG)</td></tr>
<tr><td>Y913X | p.Tyr913* (c.2739T>A)</td></tr>
<tr><td>2896insAG | p.Val922Glufs*2
(c.2763_2764dupAG)</td></tr>
<tr><td>L927P | p.Leu927Pro (c.2780T>C)</td></tr>
<tr><td>2942insT | p.Val938Glyfs*37 (c.2810dupT)</td></tr>
<tr><td>2957delT | p.Ile942Thrfs*26 (c.2825delT)</td></tr>
<tr><td>S945L | p.Ser945Leu (c.2834C>T)</td></tr>
<tr><td>2991del32 | p.Leu953Phefs*11
(c.2859_2890delACATTCTGTTCTTCAAG
CACCTATGTCAACCC)</td></tr>
<tr><td>3007delG | p.Ala959Hisfs*9 (c.2875delG)</td></tr>
<tr><td>3028delA | p.Thr966Argfs*2 (c.2896delA)</td></tr>
<tr><td>G970R | p.Gly970Arg (c.2908G>C)</td></tr>
<tr><td>G970D | p.Gly970Asp (c.2909G>A)</td></tr>
<tr><td>D979V | p.Asp979Val (c.2936A>T)</td></tr>
<tr><td>3120G->A | p.Gln996= / p.? (c.2988G>A)</td></tr>
<tr><td>3120+1G->A | p.? (c.2988+1G>A)</td></tr>
<tr><td>3121-2A->G | p.? (c.2989-2A>G)</td></tr>
<tr><td>3121-1G->A | p.? (c.2989-1G>A)</td></tr>
<tr><td>3132delTG | p.Val1001Aspfs*45
(c.3002_3003delTG)</td></tr>
<tr><td>3143del9 | p.Ala1004_Ala1006del
(c.3011_3019delCTATAGCAG)</td></tr>
<tr><td>A1006E | p.Ala1006Glu (c.3017C>A)</td></tr>
<tr><td>3171delC | p.Tyr1014Thrfs*9 (c.3039delC)</td></tr>
<tr><td>3171insC | p.Tyr1014Leufs*33
(c.3039dupC)</td></tr>
<tr><td>T1036N | p.Thr1036Asn (c.3107C>A)</td></tr>
<tr><td>Q1042X | p.Gln1042* (c.3124C>T)</td></tr>
<tr><td>3271delGG | p.? (c.3139_3139+1delGG)</td></tr>
<tr><td>3272-26A->G | p.? (c.3140-26A>G)</td></tr>
<tr><td>H1054D | p.His1054Asp (c.3160C>G)</td></tr>
<tr><td>G1061R | p.Gly1061Arg (c.3181G>C)</td></tr>
<tr><td>L1065P | p.Leu1065Pro (c.3194T>C)</td></tr>
<tr><td>R1066C | p.Arg1066Cys (c.3196C>T)</td></tr>
<tr><td>R1066H | p.Arg1066His (c.3197G>A)</td></tr>
<tr><td>3349insT | p.Tyr1073Leufs*3 (c.3217dupT)</td></tr>
<tr><td>L1077P | p.Leu1077Pro (c.3230T>C)</td></tr>
<tr><td>W1089X | p.Trp1089* (c.3266G>A)</td></tr>
<tr><td>Y1092X | p.Tyr1092* (c.3276C>A)</td></tr>
<tr><td>Y1092X | p.Tyr1092* (c.3276C>G)</td></tr>
<tr><td>W1098R | p.Trp1098Arg (c.3292T>C)</td></tr>
<tr><td>W1098X | p.Trp1098* (c.3293G>A)</td></tr>
<tr><td>W1098X | p.Trp1098* (c.3294G>A)</td></tr>
<tr><td>W1098C | p.Trp1098Cys (c.3294G>C)</td></tr>
<tr><td>W1098C | p.Trp1098Cys (c.3294G>T)</td></tr>
<tr><td>M1101K | p.Met1101Lys (c.3302T>A)</td></tr>
<tr><td>M1101R | p.Met1101Arg (c.3302T>G)</td></tr>
<tr><td>R1102X | p.Arg1102* (c.3304A>T)</td></tr>
<tr><td>E1104X | p.Glu1104* (c.3310G>T)</td></tr>
</table>

NYS Cystic Fibrosis Newborn Screening: Custom *CFTR* Variant Panel Content

Variant: Legacy	HGVS Protein (cDNA)
S1118F	p.Ser1118Phe (c.3353C>T)
3500-2A->G	p.? (c.3368-2A>G)
W1145X	p.Trp1145* (c.3435G>A)
3600G->A	p.Leu1156= / p.? (c.3468G>A)
3600+2insT	p.? (c.3468+2dupT)
3600+5G->A	p.? (c.3468+5G>A)
R1158X	p.Arg1158* (c.3472C>T)
S1159P	p.Ser1159Pro (c.3475T>C)
S1159F	p.Ser1159Phe (c.3476C>T)
**R1162X**	**p.Arg1162* (c.3484C>T)**
**3659delC**	**p.Lys1177Serfs*15 (c.3528delC)**
3667ins4	p.Thr1179Ilefs*17 (c.3532_3535dupTCAA)
S1196X	p.Ser1196* (c.3587C>G)
3737delA	p.Asp1202Alafs*9 (c.3605delA)
W1204X	p.Trp1204* (c.3611G>A)
W1204X	p.Trp1204* (c.3612G>A)
3791delC	p.Thr1220Lysfs*8 (c.3659delC)
3821delT	p.Ser1231Profs*4 (c.3691delT)

Variant: Legacy	HGVS Protein (cDNA)
I1234V	p.Ile1234Val (c.3700A>G)
3849G->A	p.Arg1239= / p.? (c.3717G>A)
3849+4A->G	p.? (c.3717+4A>G)
3849+5G->A	p.? (c.3717+5G>A)
3849+40A->G	p.? (c.3717+40A>G)
**3849+10kbC->T**	**p.? (c.3718-2477C>T)**
3850-3T->G	p.? (c.3718-3T>G)
3850-1G->A	p.? (c.3718-1G>A)
V1240G	p.Val1240Gly (c.3719T>G)
G1244E	p.Gly1244Glu (c.3731G>A)
3876delA	p.Lys1250Argfs*9 (c.3744delA)
3878delG	p.Lys1250Argfs*9 (c.3747delG)
G1249R	p.Gly1249Arg (c.3745G>A)
S1251N	p.Ser1251Asn (c.3752G>A)
L1254X	p.Leu1254* (c.3761T>G)
S1255P	p.Ser1255Pro (c.3763T>C)
S1255X	p.Ser1255* (c.3764C>A)
3905insT	p.Leu1258Phefs*7 (c.3773dupT)
I1269N	p.Ile1269Asn (c.3806T>A)

Variant: Legacy	HGVS Protein (cDNA)
**W1282X**	**p.Trp1282* (c.3846G>A)**
R1283M	p.Arg1283Met (c.3848G>T)
4005+1G->A	p.? (c.3873+1G>A)
4005+2T->C	p.? (c.3873+2T>C)
4010del4	p.Ile1295Phefs*32 (c.3883_3886delATTT)
4015delA	p.Ile1295Phefs*33 (c.3883delA)
4016insT	p.Ser1297Phefs*5 (c.3889dupT)
4022insT	p.Gly1298Trpfs*4 (c.3891dupT)
4040delA	p.Asn1303Thrfs*25 (c.3908delA)
**N1303K**	**p.Asn1303Lys (c.3909C>G)**
Q1313X	p.Gln1313* (c.3937C>T)
L1324P	p.Leu1324Pro (c.3971T>C)
Q1330X	p.Gln1330* (c.3988C>T)
L1335P	p.Leu1335Pro (c.4004T>C)
4168delCTAAGCC	p.Leu1346Metfs*6 (c.4036_4042delCTAAGCC)

Variant: Legacy	HGVS Protein (cDNA)
G1349D	p.Gly1349Asp (c.4046G>A)
4209TGTT->AA	p.Val1360Thrfs*3 (c.4077_4080delTGTTinsAA)
4218insT	p.Lys1363* (c.4086dupT)
E1371X	p.Glu1371* (c.4111G>T)
H1375P	p.His1375Pro (c.4124A>C)
4259del5	p.Leu1376Serfs*8 (c.4127_4131delTGGAT)
4279insA	p.Ile1383Asnfs*3 (c.4147dupA)
Q1382X	p.Gln1382* (c.4144C>T)
4326delTC	p.Cys1400* (c.4197_4198delCT)
Q1411X	p.Gln1411* (c.4231C>T)
Q1412X	p.Gln1412* (c.4234C>T)
4374+1G->A	p.? (c.4242+1G>A)
4374+1G->T	p.? (c.4242+1G>T)
4382delA	p.Glu1418Argfs*14 (c.4251delA)
4428insGA	p.Ser1435Glyfs*14 (c.4300_4301dupAG)

Cystic Fibrosis (CF) is screened in NYS infants at birth using a three-tier IRT-DNA-SEQ algorithm. ***Tier 1***: Immunoreactive trypsinogen (IRT) is tested in all infants. ***Tier 2***: A custom second-tier panel targeting 338 clinically-relevant *CFTR* variants is screened in infants with elevated IRT (top 5%). Variants included on the panel are listed in the table. Each is described using legacy[1] and Human Genome Variation Society (HGVS) nomenclature,[2] with cDNA nucleotide changes with respect to NCBI transcript NM_000492.3, and amino acid changes with respect to NCBI amino acid reference sequence NP_000483.3. 334 of 338 targeted variants have been classified as CF-causing by The Clinical and Functional Translation of CFTR database (CFTR2),[3,4] with the exception of R117H, a varying clinical consequence variant,[∫] and three classified as pathogenic[+] and 1 classified as likely pathogenic[§] using American College of Medical Genetics and Genomics (ACMG) standards.[5] Large deletion/duplications defined as CF-causing by CFTR2 are not included on the second-tier panel. ***Tier 3:*** Other *CFTR* variants, including pathogenic and likely pathogenic variants[5] not catalogued in CFTR2; variants of varying clinical consequence;[4] and variants of unknown[4] or uncertain significance (VOUS)[5] may be detected via expanded third-tier analysis, in which the complete *CFTR* coding sequence and other relevant regions are analyzed. Third-tier analysis is only conducted for infants with one second-tier panel variant or ultra-high IRT and no panel variants. Large deletions and duplications may be detected via third-tier analysis.

Variants recommended for population-based CF carrier screening[6] are shown in bold.

Most variants with protein effects listed as p.? represent variants that alter splicing.

[∫]Defined as a variant of varying clinical consequence by CFTR2. If R117H is detected, intron 8 polyT/TG status at c.1210-12T/c.1210-34TG is unmasked.

References

1. www.genet.sickkids.on.ca. Cystic Fibrosis Mutation Database (CFTR1). Cystic Fibrosis Centre at the Hospital for Sick Children in Toronto.
2. den Dunnen JT, Dalgleish R, Maglott DR, et al. HGVS Recommendations for the Description of Sequence Variants: 2016 Update. *Hum Mutat*. 2016;37(6):564-569.
3. Sosnay PR, Siklosi KR, Van Goor F, et al. Defining the disease liability of variants in the cystic fibrosis transmembrane conductance regulator gene. *Nat Genet*. 2013;45(10):1160-1167.
4. www.cftr2.org/index.php Release 11March2019. Clinical and Functional Translation of CFTR (CFTR2).
5. Richards S, Aziz N, Bale S, et al. Standards and guidelines for the interpretation of sequence variants: a joint consensus recommendation of the American College of Medical Genetics and Genomics and the Association for Molecular Pathology. *Genet Med*. 2015;17(5):405-424.
6. Watson MS, Cutting GR, Desnick RJ, et al. Cystic fibrosis population carrier screening: 2004 revision of American College of Medical Genetics mutation panel. *Genet Med*. 2004;6(5):387-391.
