# Supplementary material for: Validation of a Custom Next-Generation Sequencing Assay for Cystic Fibrosis Newborn Screening
Source: Int J Neonatal Screen. 2021 Nov 2;7(4):73. doi: 10.3390/ijns7040073 (PMC8628990; doi:10.3390/ijns7040073)
Supplement: Supplementary file 1 [file IJNS-07-00073-s001.zip › Supplemental File S1_Abbreviations_and_Descriptions_FINAL.pdf]

Manuscript ID: IJNS-1422004
Validation of a Custom Next-Generation Sequencing Assay for Cystic Fibrosis Newborn Screening

Supplemental File S1: List of abbreviations and descriptions.

General terms

CF - Cystic Fibrosis.

CFTR - Cystic Fibrosis Transmembrane Conductance Regulator gene.

IRT - Immunoreactive Trypsinogen. IRT is a pancreatic enzyme that can be elevated in newborns with cystic fibrosis and is used as the primary biomarker for CF newborn screening by all states in the US.

VHIRT - Very High IRT.

Next-generation sequencing (NGS) terms

BAM - Binary sequence Alignment/Map. A file generated during next-generation sequencing that contains sequence read data, generally with sequences aligned to a reference sequence.

Coverage/depth - The total number of sequencing reads for a given individual that overlap a specific region/variant.

Deduplicated - The PCR steps during library preparation for next-generation sequencing create many copies of the same input DNA molecule. Since individual DNA samples are labelled with molecular barcodes prior to amplifying regions of interest, all raw sequencing reads (or read pairs) that share the same molecular barcode can be combined into a single consensus sequence. This process is called deduplication, and calling variants using only deduplicated reads allows for high specificity variant calling because PCR errors can be corrected while generating a consensus sequence.

FASTQ - A text file generated during next-generation sequencing that contains DNA sequence data and corresponding sequence quality scores.

GSP2 - Gene Specific Primer 2. GSP2s are primers used in during the nested PCR amplification step of Anchored Multiplex PCR (**AMP**) during library preparation for next-generation sequencing.

GTF - Gene Transfer Format (also referred to as a Target Region file). A file containing GSP2 coordinates that specifies regions in which variants are reported and includes pipeline instructions.

TMF - Targeted Mutation File. A file that contains a list of specific variants for targeted variant calling using Archer's Vision algorithm, in VCF format.

VCF - Variant Call Format. A VCF file is a standardized format for storing variant information such as: chromosomal coordinates, reference allele, variant allele, and quality metrics.

Bioinformatics analysis terms

Alternate allele observation count (AO) - Number of unique molecules (i.e., sequence reads) containing the alternate allele that are used to call a variant for a given individual.

Allele fraction (AF) - The fraction of sequence reads for a given individual with the alternate allele. Ideally, heterozygous variants have an equal number of reference and alternate alleles, corresponding to an AF of 0.5.

cnv_strong_amplication_threshold - This setting in Archer Analysis for the CNV pipeline specifies the fold increase in copy number, relative to the calculated baseline, for a large duplication to be categorized as having strong evidence for a copy gain (i.e., duplication). Copy number is assessed by measuring read depth, or the number of unique sequence reads for a given individual.

cnv_strong_deletion_threshold - This setting in Archer Analysis for the CNV pipeline specifies the fold decrease in copy number, relative to the calculated baseline, for a large deletion to be categorized as having strong evidence for a copy loss (i.e., deletion). Copy number is assessed by measuring read depth, or the number of unique sequence reads for a given individual.

cnv_p_value_threshold - This setting in Archer Analysis for the CNV pipeline specifies the confidence threshold (p-value) that a copy number variant must be at or below, to be categorized as a copy gain or loss with strong evidence.

Unique fragment total - the total number of original DNA fragments extracted from a sample, i.e., after the fragmentation step of DNA library preparation, but before any PCR steps.

Unique start sites per GSP2 - The subset of unique fragments that represents the total number of unique DNA fragment lengths extracted from a sample. Since each DNA fragment is anchored by a GSP2 primer on one side, length is affected by the random start site on the opposite end of the fragment. Unique start sites can occur on multiple DNA molecules, which is why there are typically more unique fragments than unique start sites. The QC metric 'Average unique start sites per GSP2' is the average across all targeted regions.

Total AO of unique starts (UAO) - Total number of fragments with unique start sites used to call a given variant.
